# Deep-learning models for image-based gynecological cancer diagnosis: a systematic review and meta- analysis

**DOI:** 10.3389/fonc.2023.1216326

**Published:** 2024-01-11

**Authors:** Asefa Adimasu Taddese, Binyam Chakilu Tilahun, Tadesse Awoke, Asmamaw Atnafu, Adane Mamuye, Shegaw Anagaw Mengiste

**Affiliations:** ^1^ Department of Health Informatics, Institute of Public Health, College of Medicine and Health Sciences, University of Gondar, Gondar, Ethiopia; ^2^ eHealthlab Ethiopia Research Center, University of Gondar, Gondar, Ethiopia; ^3^ Department of Epidemiology and Biostatistics, Institute of Public Health, College of Medicine and Health Sciences, University of Gondar, Gondar, Ethiopia; ^4^ Department of Health Systems and Policy, Institute of Public Health, College of Medicine and Health Sciences, University of Gondar, Gondar, Ethiopia; ^5^ School of Information Technology and Engineering, Addis Ababa University, Addis Ababa, Ethiopia; ^6^ Department of Business, History and Social Sciences, University of Southeastern Norway, Vestfold, Vestfold, Norway

**Keywords:** medical image analysis, AI, deep learning, gynaecological cancer diagnosis, systematic review and meta-analysis

## Abstract

**Introduction:**

Gynecological cancers pose a significant threat to women worldwide, especially those in resource-limited settings. Human analysis of images remains the primary method of diagnosis, but it can be inconsistent and inaccurate. Deep learning (DL) can potentially enhance image-based diagnosis by providing objective and accurate results. This systematic review and meta-analysis aimed to summarize the recent advances of deep learning (DL) techniques for gynecological cancer diagnosis using various images and explore their future implications.

**Methods:**

The study followed the PRISMA-2 guidelines, and the protocol was registered in PROSPERO. Five databases were searched for articles published from January 2018 to December 2022. Articles that focused on five types of gynecological cancer and used DL for diagnosis were selected. Two reviewers assessed the articles for eligibility and quality using the QUADAS-2 tool. Data was extracted from each study, and the performance of DL techniques for gynecological cancer classification was estimated by pooling and transforming sensitivity and specificity values using a random-effects model.

**Results:**

The review included 48 studies, and the meta-analysis included 24 studies. The studies used different images and models to diagnose different gynecological cancers. The most popular models were ResNet, VGGNet, and UNet. DL algorithms showed more sensitivity but less specificity compared to machine learning (ML) methods. The AUC of the summary receiver operating characteristic plot was higher for DL algorithms than for ML methods. Of the 48 studies included, 41 were at low risk of bias.

**Conclusion:**

This review highlights the potential of DL in improving the screening and diagnosis of gynecological cancer, particularly in resource-limited settings. However, the high heterogeneity and quality of the studies could affect the validity of the results. Further research is necessary to validate the findings of this study and to explore the potential of DL in improving gynecological cancer diagnosis.

## 1 Introduction

Gynecological cancers refer to various types of cancers that affect the female reproductive system, including ovarian, cervical, uterine, vaginal, and vulvar cancers (1, 2). These cancers are a significant public health concern worldwide, with approximately 1.3 million new cases and 500,000 deaths annually (3–5). The high mortality rate associated with gynecological cancers can be attributed to late diagnosis, which underscores the importance of early detection and treatment (6).

Medical imaging, such as ultrasound, computed tomography (CT), and magnetic resonance imaging (MRI), plays a crucial role in the early detection and diagnosis of gynecological cancers. However, accurate interpretation of these images can be challenging, and traditional diagnostic methods may not always provide accurate results (7–9). In recent years, deep-learning models have emerged as a promising tool for improving the accuracy and efficiency of gynecological cancer diagnosis from medical images (10–16).

Deep-learning models are a subset of artificial intelligence (AI) that can learn to recognize patterns and features in data without being explicitly programmed. These models can be trained using large datasets of medical images to detect subtle differences between normal and abnormal tissue (17–19). Studies have shown that deep-learning models can achieve high accuracy in detecting gynecological cancers from medical images, outperforming traditional diagnostic methods such as manual interpretation by human experts (20–30).

However, the effectiveness and reliability of these models in clinical settings remain unclear, and a comprehensive review of the existing literature is necessary.

The present study aims to conduct a systematic review and meta-analysis of the current literature on deep-learning models for image-based gynecological cancer diagnosis. The review will address the following research questions:

1. What machine-learning and deep-learning models have been developed for image-based gynecological cancer diagnosis, and how do they compare in terms of performance and accuracy?
2. What are the limitations and challenges associated with the use of deep-learning models in gynecological cancer diagnosis?
3. What are the implications of the findings for the clinical application of deep-learning models in image-based gynecological cancer diagnosis?

By answering these research questions, this study provides valuable insights into the potential of deep-learning models for improving the accuracy and efficiency of gynecological cancer diagnosis and inform future research and clinical practice in this field.

## 2 Methods

### 2.1 Literature search

The literature search was conducted in accordance with the Enhancing the Quality and Transparency of Health Research (EQUATOR) Reporting Guidelines and the Preferred Reporting Items for Systematic Reviews (PRISMA-2) (31). A protocol for the study was registered in PROSPERO (ID No CRD42023421847). The search was performed on the following databases and websites: PubMed, Embase, Scopus, Google, and Google Scholar. The search was conducted from January 8 to 30, 2023, and included articles published between January 2017 and December 2022. The snowball method was used to identify relevant articles from the reference lists of retrieved articles. The sample search terms included gynecologic cancer, diagnosis, prognosis, deep learning, AI, artificial intelligence, machine learning, and neural network.

### 2.2 Inclusion and exclusion criteria

To be included in the review, studies had to meet the following criteria: (i) consideration of at least one of the five types of gynecologic cancers (cervical, ovarian, uterine, vaginal, or vulvar); (ii) use of at least one deep learning technique as a classifier; (iii) reporting of at least one performance evaluation measure for deep learning-based image segmentation of gynecologic cancers; (iv) publication between January 2017 and August 2022; (v) full-text publication in English; and (vi) availability of full-text articles. Abstracts and preprints were excluded.

### 2.3 Assessment of methodologic quality

The full texts of the selected articles were retrieved and assessed for eligibility by the same two reviewers. Two researchers reviewed (ZA and AA) the titles and abstracts of retrieved articles and applied inclusion and exclusion criteria. The full texts of qualifying articles were retrieved and reviewed to confirm study eligibility. Any disagreements were resolved by discussion or by consulting a third reviewer. The following data were extracted from each included study: authors, year of publication, country of origin, cancer type, image modality, data source, data size, data preprocessing, ML technique, performance metrics, validation method, and main findings. The quality of the studies was assessed using the Quality Assessment of Diagnostic Accuracy Studies-2 (QUADAS-2) tool, which evaluates the risk of bias and applicability of studies based on four domains: patient selection, index test, reference standard, and flow and timing. The final criterion is based on the risk of bias with respect to concerns about applicability. Rating risks of bias was determined as high, low, or unclear (32).

### 2.4 Data extraction

The following information was extracted from each included study: first author, year of publication, country of origin, cancer type, image modality, data source, data size, data preprocessing, ML technique, performance metrics, validation method, and main findings. The PRISMA guidelines were followed for data extraction (31).

### 2.5 Qualitative synthesis

A qualitative synthesis was performed to provide a narrative summary of findings from the included studies. This synthesis involved a thematic analysis to identify common themes across the literature. Reviewers independently analyzed articles, extracted key findings, and identified themes related to the use of deep learning techniques for image-based gynecological cancer diagnosis.

A constant comparative approach was used to identify similarities and differences across studies. Any discrepancies were resolved through discussion, with a third reviewer consulted when necessary. The themes identified during the qualitative synthesis were summarized and reported in the results section. The goal was to offer a comprehensive overview of the existing literature and pinpoint areas for future research.

### 2.6 Meta-analysis

A meta-analysis was performed to estimate the pooled performance of deep learning techniques for image-based gynecological cancer diagnosis. Only studies that reported sensitivity and specificity values or provided sufficient data to calculate them were included in the meta-analysis. The sensitivity and specificity values were transformed into logit values and pooled using a random-effects model. The heterogeneity of the studies was evaluated using Higgins’ I2 statistic (33). Subgroup analyses were performed based on the types of algorithm used (34). The summary receiver operating characteristic (SROC) curve and the area under the SROC curve (AUC) were calculated to summarize the overall diagnostic accuracy of advanced ML techniques. The statistical analyses were performed using R software with the “meta” and “mada” packages.

## 3 Results

### 3.1 Study selection

**Figure 1:**
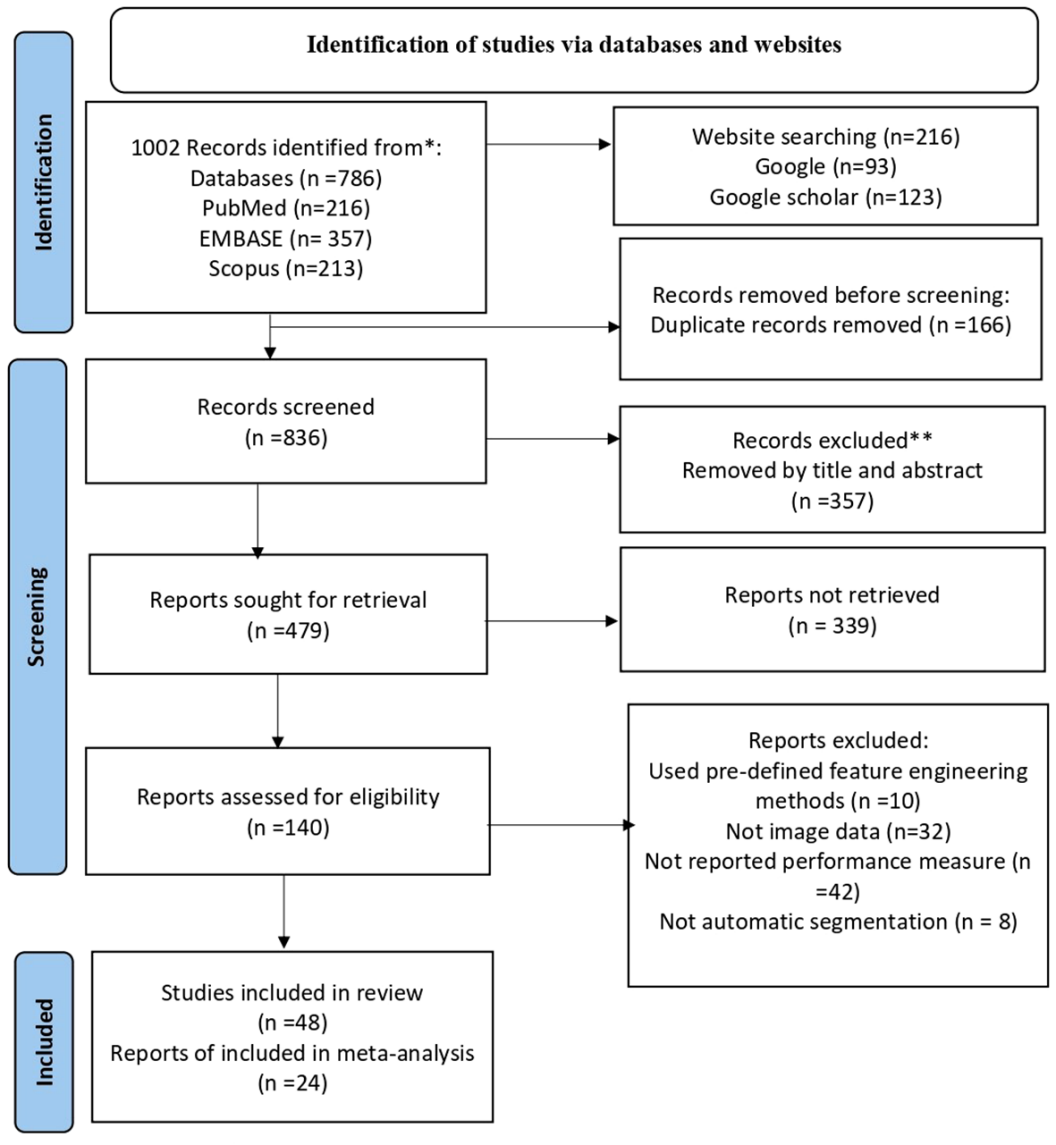
Shows the PRISMA flowchart of the sudy selection process. PRISMA stands for preferred Reporting items for Systematic Review and Meta-Analysis. He flowchart displays the search methodology and literature selection process.

The search strategy yielded 1,002 articles from the four databases. After removing duplicates, 836 articles remained for title and abstract screening. Of these, 357 were removed by title and abstract and then 339 articles were not had full texts and removed it. The remaining 140 were full manuscripts and eligible for final screening. However, 92 articles were excluded because of the absence of image data and performance measure. Finally, 48 studies were eligible and included in our systematic review and of which 24 studies were available for meta-analysis (**Figure 1**).

### 3.2 Study characteristics

A total of 48 studies were included in this analysis. The studies used various imaging modalities, including cytology (20 studies), colposcopy (15 studies), MRI (8 studies), CT scan (4 studies), and hysteroscopy (1 study). The studies were published between 2017 and 2022, with the majority (19 studies) being published in 2022. The types of cancer studied included cervical cancer (30 studies), endometrial cancer (6 studies), ovarian cancer (9 studies), combined gynecologic cancer (1 study), and vulvar and vaginal cancer (2 studies). The number of images used by literatures were ranged from 34 to 67811, with a mean of 2209 and a standard deviation of 5830. 40 studies focused on automatic image classification using various deep learning models. One study focused on the quantification of abnormalities in gynecologic cytopathology with deep learning and the remaining seven studies focused on automatic tumor segmentation using deep learning techniques (see details in **Supplementary File 1**).

### 3.3 Preprocessing and feature extraction techniques

To create deep learning models for the diagnosis of gynecologic cancer based on images, various researchers have employed distinct methodologies. Nonetheless, a consensus among the majority of authors is that initial image preprocessing, involving various techniques, is essential. Following this, the identification and extraction of crucial features are commonly performed before proceeding with post-processing steps (for additional information, please refer to **Supplementary File 2**).

The 12 articles reviewed various pre-processing and feature extraction techniques to enhance image quality and extract relevant information for analysis. These techniques were used in different combinations in the different studies. Some studies use a combination of pre-processing techniques and feature extraction methods, while others focus on one particular technique or method. For instance, Bhatt et al. (35) used techniques such as horizontal flipping, inverse rotation, random scaling, and progressive resizing to augment data in Pap smear whole slide images. Chandran et al. (36) employed techniques such as random rotation, random brightness, random crop, random blur, and max pooling to preprocess colposcopy images for the diagnosis of cervical cancer. Cheng et al. (37) used techniques such as threshold truncation, normalization, zooming, and max pooling for image preprocessing. Chen et al. (38) utilized techniques such as random rotation, oversampling, and max pooling for image preprocessing. Cho et al. (39) used techniques such as automatic central cropping, min-max normalization, data augmentation, and Test-Time Augmentations (TTA) for image preprocessing. Lastly, Dai et al. (40) used techniques such as normalization, image resizing, N4BiasFieldCorrection using ANT, and bilinear interpolation for image preprocessing in MRI images. Feature extraction methods included in the articles were Otsu model-based, Principal Component Analysis (PCA), Progressive Resizing technique, Max Pooling, t-SNE, Test-Time Augmentations (TTA), Bilinear interpolation, MobileNetv2, and Super pixel gap-search (Markov random field) (see details in **Table 1**).

**Table 1:** Indicates different pre-processing and feature extraction techniques used in the reviewed articles.

Authors (publication year)	Pre-processing technique	Feature Extraction
AbuKhalil, T., et al. (2022) (41)	Median filtering (MF)	Otsu model based
Alquran, H., et al. (2022) (42)	Combination of normalization, attribute selection, discretization, and concept hierarchy generation	Principal Component Analysis (PCA)
Best, M. G., et al. (2021) (43)	Combination of normalization, attribute selection, discretization, and concept hierarchy generation	Principal Component Analysis (PCA)
Bhatt A.R. et al. (2021) (35)	Horizontal flipping Inverse rotation Random scaling	Progressive Resizing technique
Chandran, V., et al. (2021) (36)	Random Rotation, brightness corrections, crop, blur	Max Pooling
Cheng et al (2021) (37)	Threshold Truncation, Normalization and Zooming	Max Pooling
Chen, X., et al. (2020) (38)	Random Rotation and Oversampling	Max Pooling
Cheng, W., et al. (2019) (44)	Quintile Normalization	t-SNE
Cho, B., et al. (2020) (39)	Automatic central cropping, min–max normalization, data augmentation	Test-Time Augmentations (TTA)
Dai , M., et al. (2022) (40)	Normalization, Resizing	N4BiasFieldCorrection Bilinear interpolation
Habtemariam, L. et al. (2022) (45)	Histogram Matching Image-to-Image Translation Real-time augmentation	MobileNetv2
Kudva, V. and K. Prasad (2020) (46)	RGB channel superposition Bi-Histogram Equalization with adaptive sigmoidal function combined with sobel operator (horizontal and vertical)	Super pixel gap-search (Markov random field) Max pooling

### 3.4 Deep learning models in gynecologic cancer diagnosis

**Figure 2:**
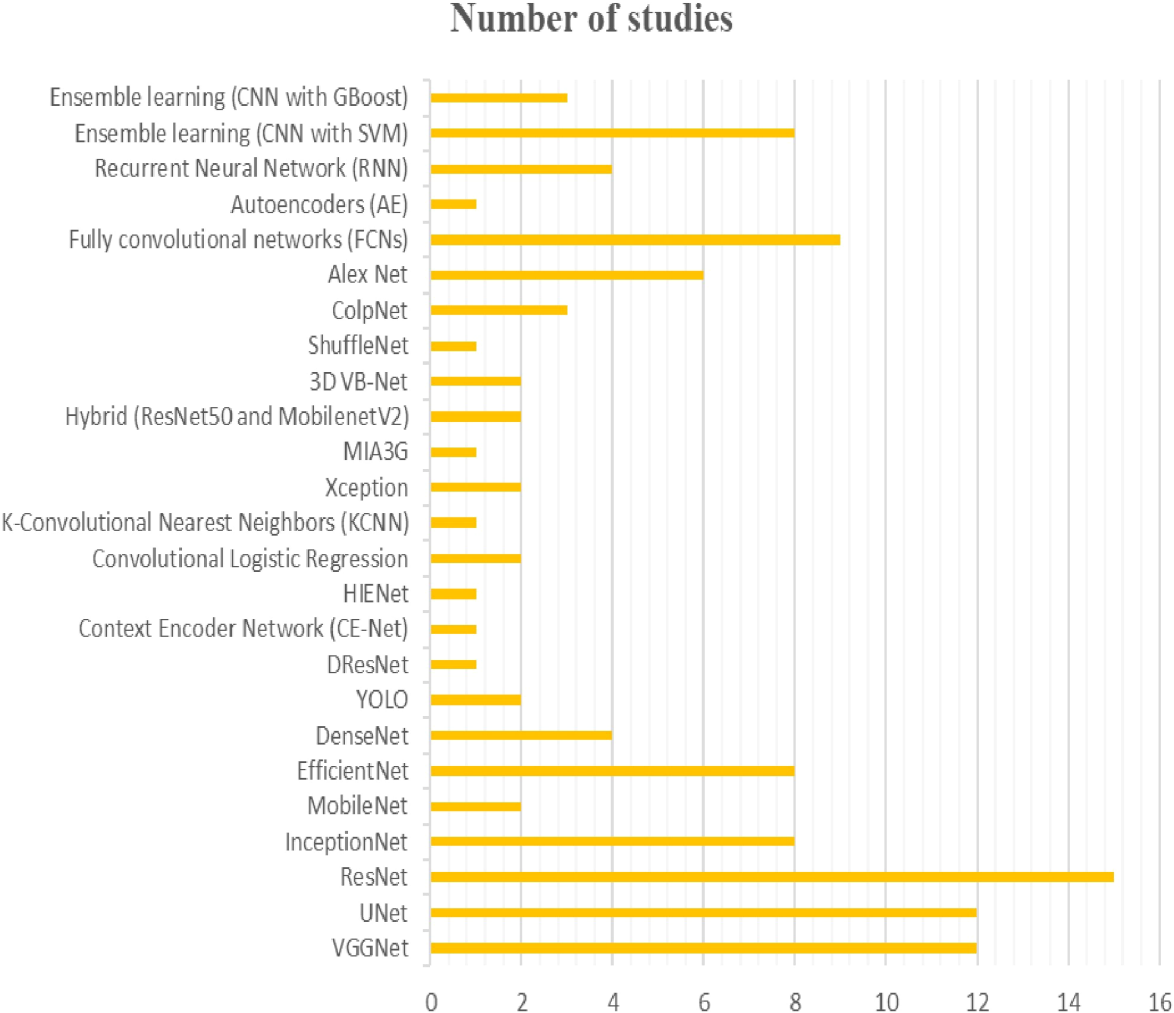
The figure provides an overview of the diversiy and popularity of models used in different studies for the diagnosis of gynecologic cancers.

In the context of gynecologic cancer diagnosis, deep learning models have been widely used. Many studies have employed convolutional neural networks (CNNs) or their variants, such as VGGNet, UNet, ResNet, InceptionNet, MobileNet, EfficientNet, DenseNet, YOLO, DResNet, CE-Net, HIENet, Xception, MIA3G, Hybrid, 3D VB-Net, ShuffleNet and ColpNet. Other models, such as autoencoders (AE), recurrent neural networks (RNN) and ensemble learning methods (CNN with SVM or XGBoost), have also been utilized. Among these models, ResNet is the most popular (appearing in 15 studies), followed by VGGNet and UNet (appearing in 12 studies each), InceptionNet and EfficientNet (appearing in 8 studies each), and DResNet, CE-Net, HIENet, KCNN, and MIA3G (appearing in only one study each) (see **Figure 2**).

### 3.5 Deep learning for gynecologic cancer segmentation

Seven studies that used different deep learning models, including 3D-UNet, 3D VB-Net, ResNet18, 2D-RefineNet, CE-Net, and fully convolutional neural networks, were reviewed for gynecologic cancer segmentation. The studies also used different types of images (CT or MRI) and had various sample sizes, ranging from 130 to 826 images. Various model performance measures were reported, such as the 95% Hausdorf distance (HD_95), Dice Similarity Coefficient (DSC), Mean Surface Distance (MSD), Jaccard index (JI), and Average Surface Distance (ASD).

The DSC score, which measures the overlap between automatic and manual segmentation, was one of the most commonly used performance measures. Cheng et al. (37) achieved the highest DSC score for CTV segmentation using 3D-UNet on 400 MRI images, with a score of 0.93. For bladder segmentation, Ding et al. (47) and Cheng et al. (37) achieved the highest DSC score using 3D-UNet on 130 and 400 MRI images, respectively, with a score of 0.91. Ma et al. (48) achieved the highest DSC score for rectum segmentation using 3D VB-Net on 200 CT images, with a score of 0.88. Ding et al. (47) also achieved the highest DSC score for femoral head segmentation using 3D VB-Net on 130 MRI images, with a score of 0.92.

The HD_95, which measures the maximum distance between automatic and manual segmentation boundaries, was another commonly used performance measure. Ding et al. (47) achieved the lowest HD_95 for CTV segmentation using 3D-UNet on 130 MRI images, with a score of 10.03. Ma et al. (48) achieved the lowest HD_95 for bladder and rectum segmentation using 3D VB-Net on 200 CT images, with scores of 4.86 and 4.11, respectively. Ma et al. (48) also achieved the lowest HD_95 for femoral head segmentation using 3D VB-Net on 302 CT images, with a score of 4.86(see details in **Table 2**).

**Table 2:** Indicates different deep learning for gynecologic cancer segmentation.

Authors (publication year)	Number of Images	Advanced ML models	Model performance measures				
			95% Hausdorf distance (HD_95)	DSC	MSD (mm)	Jaccard index (JI)	Average surface distance (ASD)
Cheng et al. (2021) (37)	400	3D-UNet	10.03	85.2			
Ding, Y., et al. (2022) (47)	130	3D-UNet	10.08	85		77	2.58
		3D VB-Net	11.2	83		75	2.26
Lin, Y., et al. (2020) (49)	169	ResNet18		82			
Ma, C., et al. (2022) (48)	200	3D VB-Net	4.86	88	1.32		
	335	3D VB-Net	6.47	70	2.42		
	302	3D VB-Net	4.11	86	1.15		
Williams, M., et al. (2018) (50)	169	3D-UNet		82			
Xiao, C., et al. (2022) (51)	313	2D-RefineNet		82			
		Fully convolutional neural networks		80			
		2D-UNet		82			
		Context Encoder Network (CE-Net)		81			
		3D-UNet		80			
		3D- ResUNet		81			
		3D-RefineNet		82			
Zaffino, P., et al. (2022) (52)	826	3D-UNet		60	2		

### 3.6 Deep learning models used for abnormality detection

**Figure 3:**
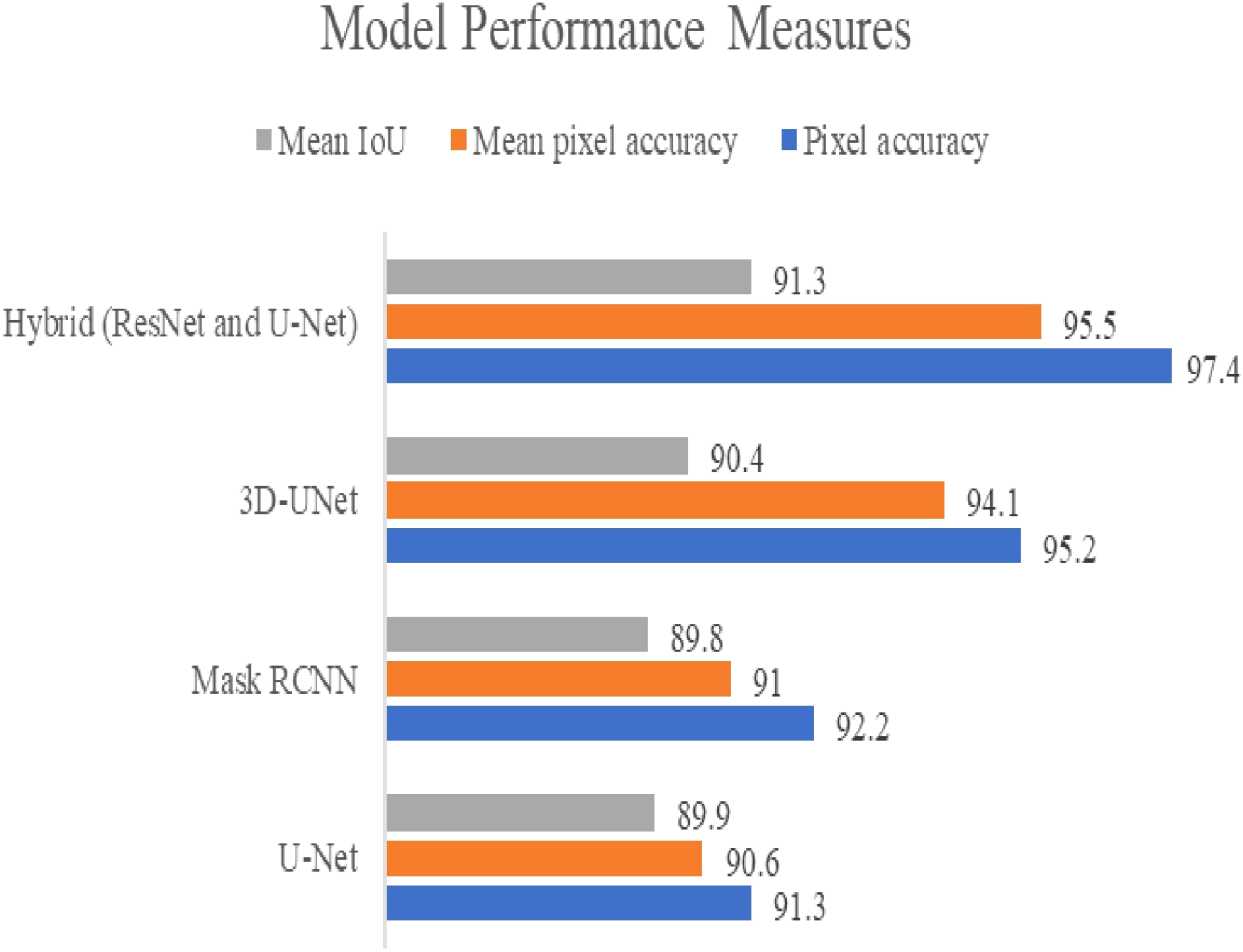
Showed deep learning models used to quantify images.

Several models have been utilized by Ke and Shen for automatic abnormality detection from medical images, including U-Net, Mask RCNN, 3D-UNet, and a ResNet-U-Net hybrid. Performance metrics such as pixel accuracy, mean pixel accuracy, and mean IoU were used to evaluate the models, with the ResNet-U-Net hybrid achieving the highest performance, scoring 97.4%-pixel accuracy, 95.5% mean pixel accuracy, and 91.3% mean IoU. On the other hand, U-Net had the lowest performance, with a pixel accuracy of 91.3%, mean pixel accuracy of 90.6%, and mean IoU of 89.9%. **Figure 3** displays the deep learning models utilized for quantifying images (See **Figure 3**).

### 3.7 Deep learning models used for automatic image classification

In the literature reviewed for gynecologic cancer screening and diagnosis, various deep learning models were employed, including ResNet50, Colponet, ResNeSt, N-Net, 3D-UNet, and YOLOv3. Several studies reported high performance measures, with ResNet-v2 used by AbuKhalil et al. (41) achieving 96.7% precision, 97.39% sensitivity, and 96.61% accuracy on 918 images. Bhatt et al. (35) utilized convNet with transfer learning and progressive resizing with K-Nearest Neighbour and EfficientNet-B3 on 917 and 966 images, respectively, achieving 78.14% and 99.01% accuracy (**Table 3** for details).

**Table 3:** The table provides an overview of the performance and diversity of deep learning models used in different studies for gynecologic cancer screening and diagnosis.

Authors (Publication year)	No of Images	Focus	Advanced ML Models	Performance measures
AbuKhalil, T., et al. (2022) (41)	918	Optimal Deep Learning Based Inception Model for Cervical Cancer Diagnosis	ResNet- v2	Precision = 96.7 Sensitivity = 97.39 Accuracy = 96.61
Best, M. G., et al. (2021) (43)	5,271	CAD system based on deep learning for classifying colposcopy images.	ResNet-50	Accuracy = 88.2 Specificity = 90.1 PPV (%) =86.7 NPV (%) = 83.8 AUC = 93.6
Bhatt AR.et al. (2021) (35)	917	Cervical cancer detection in pap smears whole slide images using convNet with transfer learning and progressive resizing.	K-Nearest Neighbour	Precision = 79.27 Sensitivity = 95.59 Accuracy = 78.14 F1-score =86.67
Bhatt AR. et al. (2021) (35)	966	Cervical cancer detection in pap smears whole slide images using convNet with transfer learning and progressive resizing.	EfficientNet-B3	Precision = 99.15 Sensitivity = 98.89 Accuracy = 99.01 Specificity = 99.02 F1-score = 98.87
Chandran, V., et al. (2021) (36)	5679	Diagnosis of Cervical Cancer based on Ensemble Deep Learning Network using Colposcopy Images	CYENET	Sensitivity = 92.4 Accuracy = 92.3 Specificity = 96.2 PPV (%) = 92 NPV (%) = 95
Cheng, S., et al. (2021) (37)	3545	Robust whole slide image analysis for cervical cancer screening using deep learning	Recurrent Neural Network (RNN)	Sensitivity = 81.9 Accuracy = 89 Specificity = 79.3
Cho, B. J., et al. (2020) (39)	791	Classification of cervical neoplasms on colposcopy photography using deep learning	Resnet-152	Sensitivity = 85.2 Accuracy = 87.7 Specificity = 88.2 PPV (%) = 58.9 NPV (%) = 97
Cho, B. J., et al. (2022) (53)	588	Automated Diagnosis of Cervical Intraepithelial Neoplasia in Histology Images via Deep Learning	DenseNet-161	Accuracy = 93.2 AUC = 0.99
Habtemariam, L., et al. (2022) (45)	4005	Cervix Type and Cervical Cancer Classification System Using Deep Learning Techniques	ResNet50	Accuracy = 90.73
Hou, X., et al. (2022) (54)	34	Artificial Intelligence in Cervical Cancer Screening and Diagnosis	EfficienetNetB0	Accuracy = 92.19
Karasu Y., et al. (2022) (55)	2452	Deep Learning Models for Automated Cross-Preparation Diagnosis of Multi-Cell Liquid Pap Smear Images	ResNet50	Precision = 99.2 Sensitivity = 99 Accuracy = 99.19 AUC = 0. 99 F1-score =99.19
Saini, B. et al., (2020) (56)	800	Automated cervical cancer screening using colposcopy images	Colponet	Accuracy = 83.4
Nambu, Y. et al. (2022) (57)	919	A screening assistance system for cervical cytology of squamous cell atypia based on a two-step combined CNN algorithm with label smoothing	ResNeSt	Precision = 71.89 Sensitivity = 70.8 Accuracy = 90.5
Park, Y. R., et al. (2021) (58)	4119	Comparison of machine and deep learning for the classification of cervical cancer based on cervicography images	ResNet50	Precision = 93.9 Sensitivity = 89 Accuracy = 91 AUC = 0.97 F1-score =91
Sheikhzadeh F., et al. (2018) (59)	749	Automatic labelling of molecular biomarkers of immunohistochemistry images using fully convolutional networks	N-Net	Accuracy = 92 F1-score =96
Wang, H., et al. (2022) (60)	917	Recognition and Clinical Diagnosis of Cervical Cancer Cells Based Lightweight Deep Network for Pathological Image	ResNet-50	Accuracy = 95.4
Williams, M., et al. (2018) (50)	169	Deep learning for fully automated tumor segmentation and extraction of MRI features in cervical cancer	3D-UNet	Sensitivity = 89 PPV (%) = 92
Chen, X., et al. (2020) (38)	530	Deep learning for the determination of myometrial invasion depth and automatic lesion identification in endometrial cancer MR imaging	You Only Look Once v3 (YOLOWv3)	Precision = 86.67 Sensitivity = 66.78 Accuracy = 84.7 Specificity = 87.5 PPV (%) = 44.44 NPV (%) = 94.59
Dai, M., et al. (2022) (40)	86	Application of machine learning for the differentiation of uterine sarcomas from atypical leiomyoma’s	3D-Unet	Precision = 87 Sensitivity = 76 Accuracy = 77
Kudva, V. et al. (2019) (61)	2198	Transfer Learning for Classification of Uterine Cervix Images for Cervical Cancer Screening	AlexNet	Sensitivity = 93.3 Accuracy = 93.4 Specificity = 93.2
Kudva , V. et al (2020) (46)	1644	Hybrid Transfer Learning for Classification of Uterine Cervix Images for Cervical Cancer Screening	Hybrid (AlexNet and VGG-16)	Sensitivity = 89.16 Accuracy = 91.46 Specificity = 93.83
Sun, H., et al. (2020) (62)	3302	CAD in Histopathological Images of the Endometrium Using a Convolutional Neural Network and Attention Mechanisms	HIENet	Precision = 96.7 Sensitivity = 81.04 Accuracy = 93.53 Specificity = 94.78 AUC = 0.96
Urushibara, A., et al. (2022) (63)	618	The efficacy of deep learning models in the diagnosis of endometrial cancer using MRI: a comparison with radiologists	Fully Convolutional Neural Network (CNN)	Sensitivity = 80 Accuracy = 80 Specificity = 80 AUC = 0.87
Zhang Z., et al. (2021) (64)	1851	Deep learning model for classifying endometrial lesions	VGG-16	Precision = 93.3 Sensitivity = 83 Accuracy = 90.8 Specificity = 96 AUC = 0.94 F1-score = 87.8
Takahashi Y., et al., (2021) (65)	600	Automated system for diagnosing endometrial cancer by adopting deep-learning technology in hysteroscopy	ResNet50	Precision = 83 Sensitivity = 82 Accuracy = 91 Specificity =72

In this review, ResNet, a commonly used CNN network, has demonstrated effectiveness in gynecologic cancer tasks like cervical cancer detection, endometrial cancer diagnosis, and ovarian cancer classification. ResNets utilize skip connections, enabling the network to learn identity mappings, preventing overfitting. ResNets consist of residual blocks with convolutional layers, batch normalization, and activation functions. Skip connections can be implemented using identity or projection shortcuts, impacting network performance (66) (see **Supplementary File 3** for detail).

### 3.8 Pooled performance of DL algorithms

**Figure 4:**
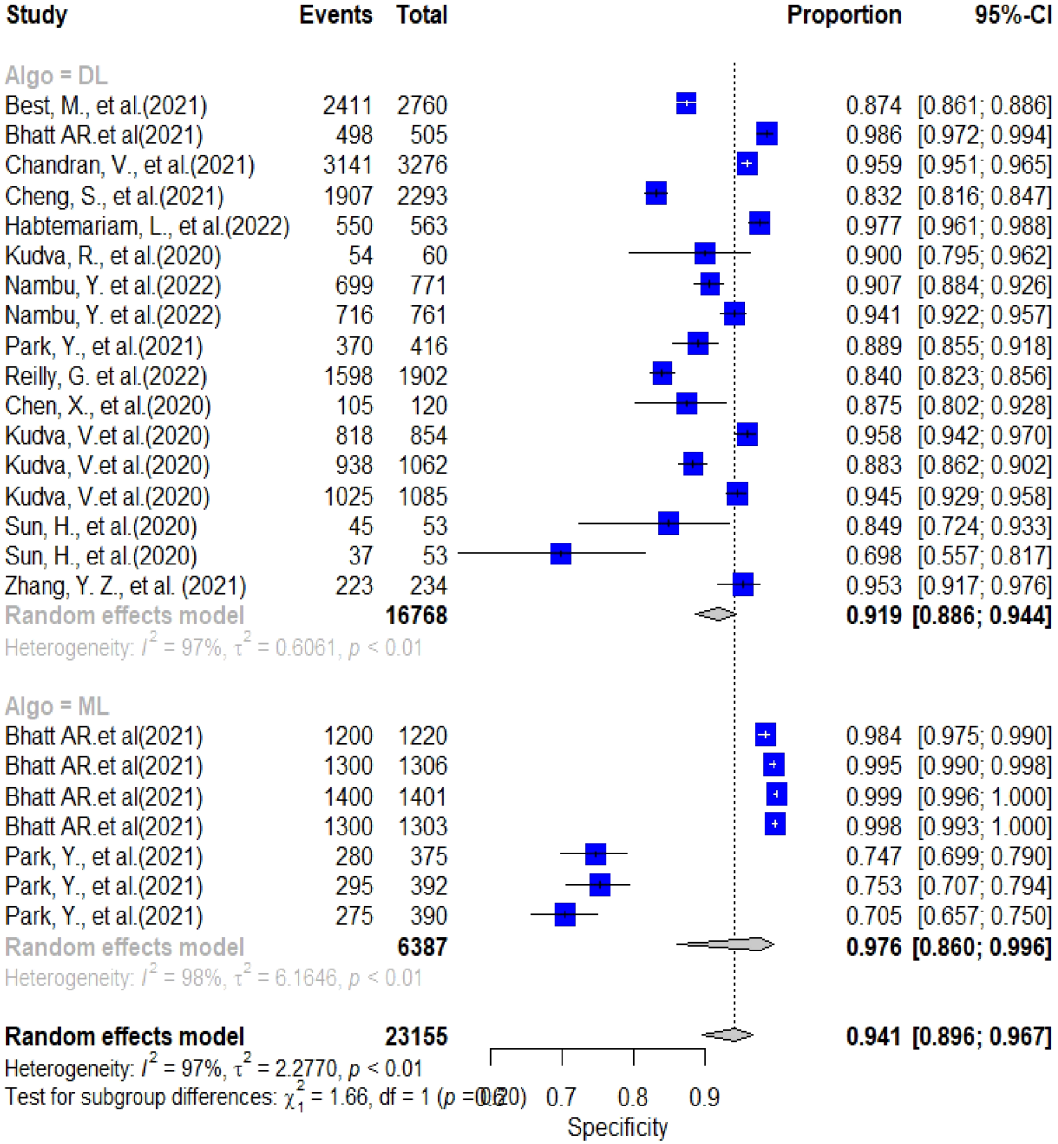
Summary estimate of pooled specificity of 24 studies using forest plot.

**Figure 5:**
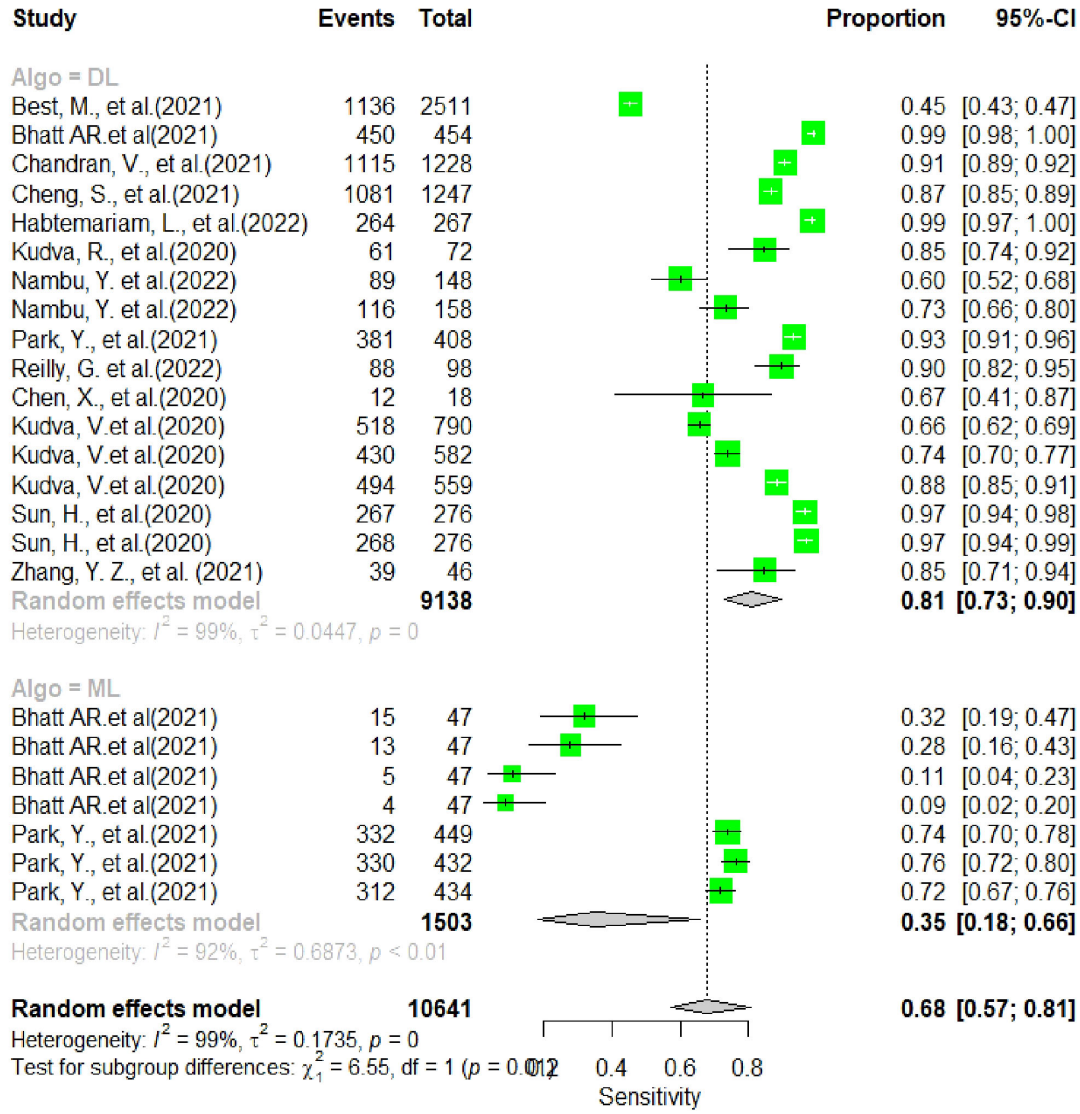
Summary estimate of pooled sensitivity of 24 studies using forest plot.

In this section, the researchers analyzed 48 studies that applied deep learning (DL) algorithms for diagnosing gynecologic cancer. However, only 24 of these studies had sufficient data to calculate the diagnostic accuracy using contingency tables. The authors used hierarchical summary receiver operating characteristic (SROC) curves to summarize the overall performance of the DL algorithms across the studies. The sensitivity and specificity were plotted for each study, with sensitivity measuring the proportion of true positives and specificity measuring the proportion of true negatives. The area under the curve (AUC) was used as a measure of the overall accuracy of the algorithm. The results showed that the pooled sensitivity and specificity for all DL algorithms were 89.40% (95% CI, 86.19–92.62%) and 87.6% (95% CI, 82.6–92.46%), respectively, with an AUC of 0.88 (95% CI, 0.84–0.93). Some studies used more than one DL algorithm and reported the best accuracy among them. The authors also summarized the performance of the best DL algorithms across the studies, with pooled sensitivity and specificity of 68.1% (95% CI, 57.2–80.9) and 94.1% (95% CI, 89.6–96.7), respectively, and an AUC of 0.81 (95% CI, 0.90–0.94). These results demonstrate that DL algorithms have a high diagnostic accuracy for gynecologic cancer (See **Figures 4**, **5** for details).

### 3.9 Subgroup meta-analyses

**Figure 6:**
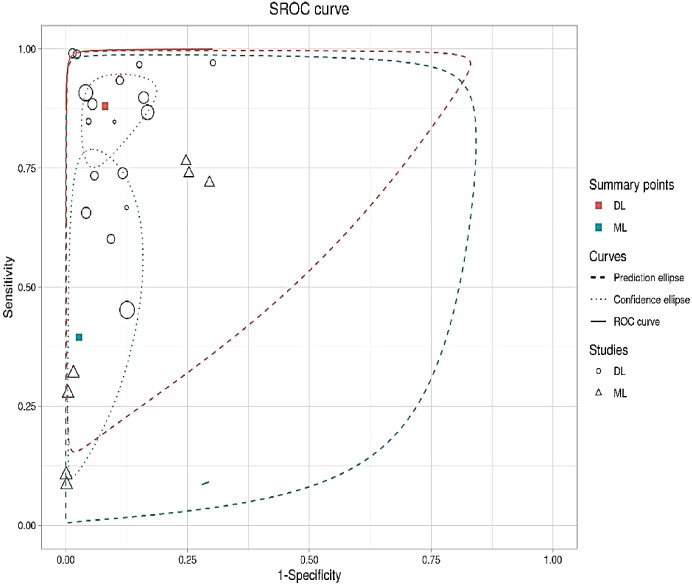
Pooled performance of DL algorithms versus ML using the same sample.

In this analysis, 24 studies were used to compare the performance of deep learning (DL) algorithms and machine learning (ML) methods in diagnosing gynecologic cancer. A random model was utilized to calculate the pooled sensitivity and specificity for each algorithm type. The results showed that DL algorithms had higher sensitivity (80% [95% CI, 73.1 – 89.7%]) and lower specificity (91.9% [88.6 – 94.4%]) compared to ML methods (sensitivity: 34.6% [95% CI, 18.2-65.8%]; specificity: 97.6% [95% CI, 86.0- 99.6%]). Additionally, the area under the curve (AUC) was higher for DL algorithms (0.86 [0.81–0.92]) than for ML methods (0.66 [0.52–0.83]), which is a measure of the overall accuracy of the algorithm. These findings suggest that DL algorithms perform better than ML methods in detecting gynecologic cancer (See **Figure 6** for details).

### 3.10 Heterogeneity analysis

**Figure 7:**
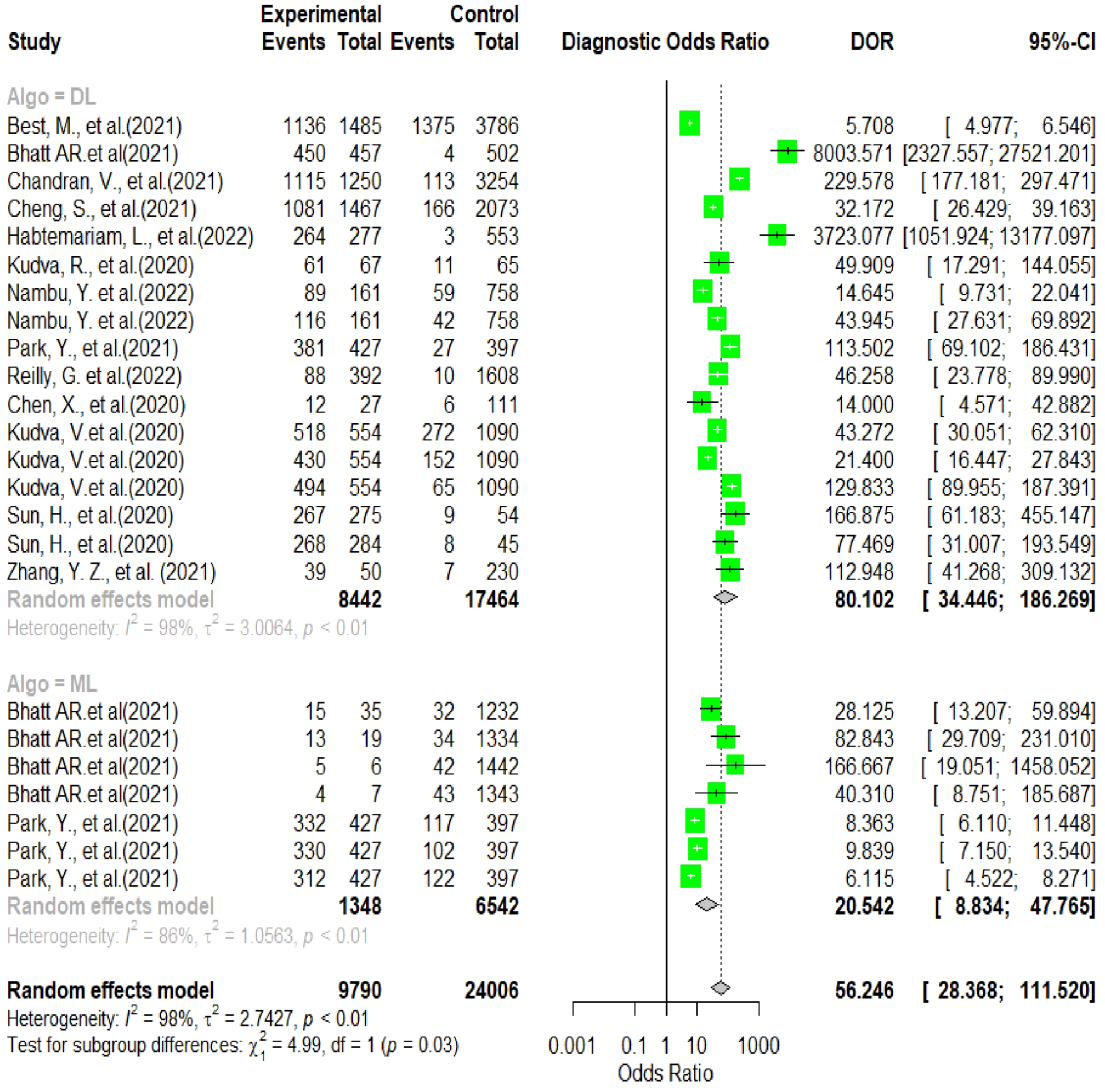
Summary of pooled odds ratio of 24 studies using forest plot.

**Figure 8:**
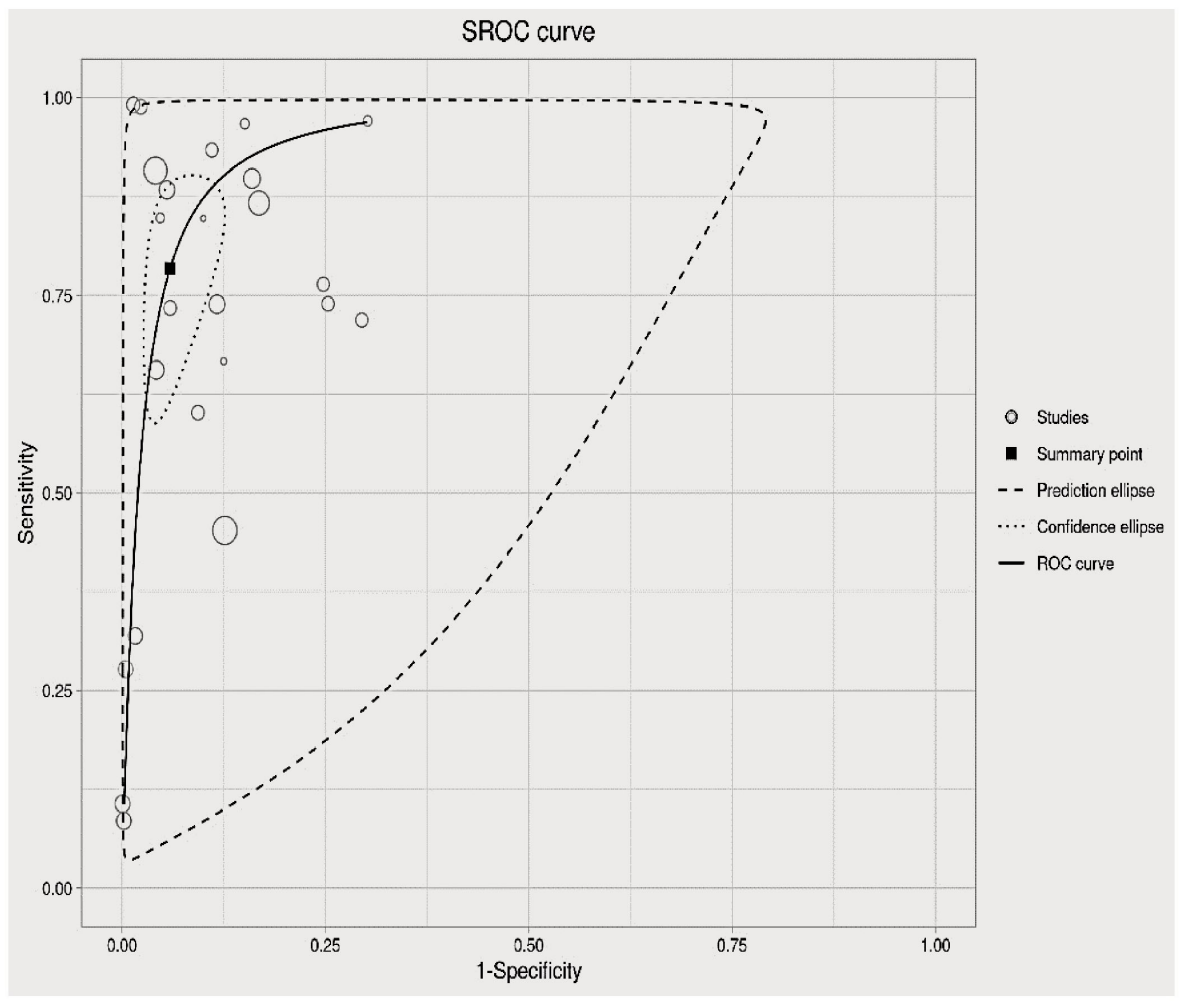
Summary of the receiver operating characteristic (SROC) plot of the advanced machine learning algorithms.

The analysis of heterogeneity among studies comparing deep learning (DL) algorithms and traditional machine learning (ML) approaches for gynecologic cancer detection revealed a high degree of variability (I2 = 98.1%, p < 0.0001). Using the inverse variance method and the DerSimonian-Laird estimator, the pooled sensitivity (SE) and specificity (SP) for the two types of algorithms were calculated. The results showed that DL algorithms had a significantly higher SE (98.9% [98.7%; 99.1%]) than ML methods (34.6% [95% CI, 18.2-65.8%]), while ML methods had a higher SP (97.6% [95% CI, 86.0- 99.6%]) than DL algorithms (91.9% [88.6 – 94.4%]). The area under the curve (AUC) was also found to be higher for DL algorithms (0.86 [0.81–0.92]) than for ML methods (0.66 [0.52–0.83]), indicating that DL algorithms are more accurate at detecting gynecologic cancer. These findings highlight the potential of DL algorithms in improving the accuracy of gynecologic cancer diagnosis, but also call for further investigation to address the heterogeneity observed among studies (see **Figure 5** for detail). The results of our analysis show that deep learning (DL) algorithms are significantly more effective than traditional machine learning (ML) approaches when it comes to correctly classifying patients with gynecologic cancer. Specifically, the pooled odds ratio (OR) of the random effect model was found to be 56.2459 [95% CI, 28.3682; 111.5195), with a p-value less than 0.0001. This means that DL algorithms are approximately 56 times more likely than ML methods to make accurate diagnoses of gynecologic cancer. These findings have important implications for the development of diagnostic tools and the treatment of patients with gynecologic cancer, suggesting that DL algorithms may offer a more effective and reliable approach to diagnosis and treatment (See **Figure 7** for details). In this study, the authors used advanced deep learning models to diagnose gynecologic cancer based on various data types. The SROC plot showed that the deep learning models had a high sensitivity and specificity for gynecologic cancer diagnosis. The AUC was 0.81, which indicates a good performance (See **Figure 8** for details).

### 3.11 Quality assessment

**Figure 9:**
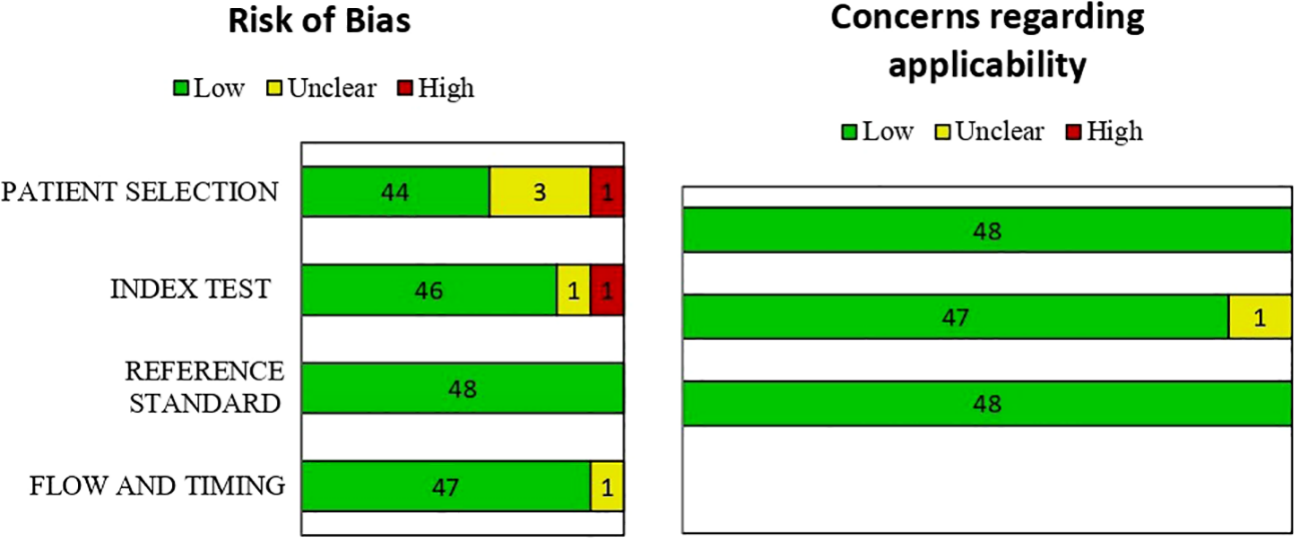
Risk of bias and concern of applicability for each item in included studies.

We used QUADAS-2 to evaluate the quality of the studies and presented a summary of findings with a suitable diagram in **Figure 9**. Out of 48 studies, 41 had low risk of bias and 7 had high or unclear risk of bias. Four studies had high or unclear risk of bias in the patient selection domain because they did not report their inclusion or exclusion criteria or they excluded patients improperly. Two studies had high or unclear risk of bias in the index test domain because they did not have a predefined threshold (see **Figure 9**).

## 4 Discussion

The studies included in this review showed a great diversity in terms of imaging modalities, publication years, types of cancer, number of images, and deep learning applications. The studies were mostly recent, with almost half of them being published in 2022. Cervical cancer was the most common type of cancer studied, followed by ovarian cancer and endometrial cancer. Gynecologic cancer and vulvar and vaginal cancer were the least common types of cancer studied. The number of images used in the studies varied widely, from a few dozens to tens of thousands. The majority of the studies focused on automatic image classification using deep learning models, such as convolutional neural networks, recurrent neural networks, or attention mechanisms. Only a few studies focused on quantification or segmentation of gynecologic abnormalities using deep learning techniques.

Cytology and colposcopy were the most common imaging modalities used, followed by MRI and CT scan. Hysteroscopy was the least common modality used. Cytology is a simple, inexpensive, and widely available method for screening and diagnosing cervical cancer. However, it has low sensitivity and specificity, especially for high-grade lesions and adenocarcinoma. It also requires adequate sampling and interpretation by trained personnel. Cytology alone is not sufficient for staging cervical cancer or detecting recurrence (67). Colposcopy is a visual examination of the cervix using a magnifying device. It can identify abnormal areas that may need biopsy or treatment. It can also assess the extent of cervical lesions and guide conization or excision procedures. However, colposcopy is operator-dependent and subjective. It may miss lesions in the endocervical canal or outside the transformation zone. It also has limited value for staging cervical cancer or detecting recurrence (67). MRI is regarded as the gold standard for local staging of most gynecologic malignancies (68). It has superb soft tissue contrast and resolution without exposing the patients to ionizing radiation. It can delineate tumor size, depth of invasion, parametrial involvement, lymph node status, and distant metastasis. Advances in functional MRI with diffusion-weighted and dynamic contrast-enhanced sequences provide more detailed information regarding tumor cellularity, vascularity, and viability (69). However, MRI is expensive, time-consuming, and not widely available. It may also have artifacts or false-positive findings due to inflammation, fibrosis, or post-treatment changes (68).

CT scan is a fast and widely available imaging modality that can evaluate the whole abdomen and pelvis in one examination. It can detect enlarged lymph nodes, ascites, peritoneal implants, liver metastasis, and other signs of advanced disease. However, CT scan has low sensitivity and specificity for local staging of gynecologic cancers. It also exposes the patients to ionizing radiation (69).

This review revealed that various pre-processing and feature extraction techniques were applied to gynecologic cancer image analysis in different studies. The most common pre-processing techniques used by different authors are filtering, normalization data augmentation and histogram matching. Filtering is a technique used to remove noise and artifacts from the images, such as Gaussian noise, speckle noise, or motion blur. It can be done using different methods, such as average filter, median filter, adaptive median filter, or Gaussian filter (70, 71). Normalization technique can adjust the intensity values of the images to a common scale, such as 0-1 or 0-255. It helps to reduce the effect of illumination variations and enhance the contrast of the images (70, 72). Data augmentation is a technique of generating new images from the existing ones by applying transformations, such as rotation, flipping, scaling, cropping, or zooming. It helps to increase the size and diversity of the dataset and reduce the overfitting problem (70, 72). And also, Histogram matching is a pre-processing technique used to modify the histogram of an image to match the histogram of another image. It helps to improve the quality and consistency of the images and reduce the effect of scanner variations (72).

Principal Component analysis (PCA), max pooling, t-distributed stochastic neighbor embedding (t-SNE) and Test-time augmentations (TTA) techniques are widely used feature extraction techniques in the literature. Principal component analysis (PCA can reduces the dimensionality of the data by projecting it onto a lower-dimensional subspace that captures most of the variance (71). Max pooling reduces the size of the feature maps by applying a max operation over a sliding window and it can helps to extract the most salient features and make them invariant to small translations (71). T-distributed stochastic neighbor embedding (T-SNE) can reduces the dimensionality of the data by embedding it into a lower-dimensional space that preserves the local similarities. T-SNE can help visualize and cluster high-dimensional data. Test-time augmentations (TTA) technique applies data augmentation techniques at test time and averages the predictions from multiple augmented images and it helps to improve the robustness and accuracy of the classification.

Our results also showed that 3D VB-Net achieved the best performance among the DL models for gynecological cancer segmentation. The 3D VB-Net model had an average HD_95 of 5.48 mm, which means that 95% of the distances between the predicted and ground truth boundaries were less than 5.48 mm. The model also had an average DSC of 81%, which means that the overlap between the predicted and ground truth regions was 81%. The model had an average MSD of 1.63 mm, which means that the average distance between the predicted and ground truth centroids was 1.63 mm. Finally, the model had an average JI of 75%, which means that the ratio of the intersection and union of the predicted and ground truth regions was 75%. These metrics indicate that the 3D VB-Net model was able to segment the gynecological tumors accurately and consistently. The worst performance was obtained by ResNet18, which had an average DSC of only 82%. This means that the ResNet18 model had a lower overlap between the predicted and ground truth regions than the other models. The other models had similar performance, with average HD_95 ranging from 10.03 to 11.2 mm, DSC from 80 to 85%, MSD from 1.15 to 2.58 mm, and JI from 75 to 77%. These metrics indicate that the other models were able to segment the gynecological tumors reasonably well, but not as well as the 3D VB-Net model. Our findings are consistent with previous studies that reported superior performance of 3D VB-Net over other DL models for prostate cancer segmentation (64, 73, 74). The advantages of 3D VB-Net include its ability to capture volumetric information from MRI images, which is important for tumor detection and characterization. The model also uses a Variational auto encoder for feature extraction, which is a generative model that can learn a latent representation of the data and reconstruct it with minimal error. The model also incorporates boundary loss for accurate segmentation, which is a loss function that penalizes the deviation of the predicted boundaries from the ground truth boundaries (64). On the other hand, ResNet18 performed poorly in our study, which might be due to its shallow architecture and lack of spatial information (49). ResNet18 is a convolutional neural network that has only 18 layers, which might not be enough to learn complex features from MRI images. The model also does not use any spatial information, such as coordinates or distances, which might be useful for tumor localization and segmentation.

The best performance of models for gynecologic cancer classification was obtained by Bhatt et al. (35) who used EfficientNet-B3, which is a convolutional neural network that uses a compound scaling method to balance the depth, width, and resolution of the network. They trained their model on 966 images of patients with cervical cancer and evaluated it on 101 images of patients with benign or malignant lesions. They achieved an accuracy of 99.01%, which means that they correctly classified 99.01% of the lesions as benign or malignant. They also achieved a precision of 99.15%, which means that 99.15% of the lesions that they predicted as malignant were actually malignant. They achieved a sensitivity of 98.89%, which means that they detected 98.89% of the malignant lesions in the dataset. They achieved a specificity of 99.02%, which means that they rejected 99.02% of the benign lesions in the dataset. They achieved an F1-score of 98.87%, which is a harmonic mean of precision and sensitivity that measures the balance between them. Another model that achieved a high performance for gynecologic cancer classification was obtained by Dai et al. (40) who used 3D-UNet, which is a deep convolutional neural network that can segment 3D volumes. They trained their model on 86 MRI images of patients with cervical or ovarian cancer and evaluated it on 43 MRI images of patients with benign or malignant tumors. They achieved an accuracy of 94.19%, which means that they correctly classified 94.19% of the tumors as benign or malignant. They also achieved a precision of 95.35%, which means that 95.35% of the tumors that they predicted as malignant were actually malignant. They achieved a sensitivity of 93.02%, which means that they detected 93.02% of the malignant tumors in the dataset. They achieved a specificity of 95.35%, which means that they rejected 95.35% of the benign tumors in the dataset. They achieved an F1-score of 94.19%, which is a harmonic mean of precision and sensitivity that measures the balance between them. They achieved an AUC of 0.94, which is the area under the receiver operating characteristic curve that measures how well the model can distinguish between benign and malignant tumors. Our findings are consistent with previous studies that reported the benefits of CNNs with transfer learning and progressive resizing for cervical cancer detection (75) and 3D-UNet or its variants for ovarian cancer detection (52, 54, 76). The advantages of these models include their ability to learn high-level features from medical images, such as edges, shapes, textures, and patterns that are relevant for tumor identification and characterization. They also have the ability to adapt to different domains and modalities, such as histopathology, ultrasound, or MRI, by transferring the knowledge learned from one domain or modality to another. They can also handle large-scale and imbalanced datasets, such as those with more benign than malignant tumors or vice versa, by using data augmentation techniques or class weighting schemes to increase the diversity and balance of the data. Moreover, they can improve segmentation accuracy by using 3D information from MRI images, such as depth and volume, and by using loss functions that emphasize the boundary accuracy, such as dice loss or focal loss.

Other studies have also used deep learning models and techniques for gynecologic cancer diagnosis using different types of images. For example, Gao et al. (2021) used a deep convolutional neural network (DCNN) to diagnose ovarian cancer using multimodal medical images (FDG-PET/CT) with an accuracy of 0.94 and an AUC of 0.98 (77). Ho et al. (2022) used deep interactive learning to diagnose BRCA mutation status in ovarian cancer using H&E-stained whole slide images with an accuracy of 0.86 and an AUC of 0.91 (78). Li et al. (79) used a self-adapting ensemble method to diagnose gynecological brachytherapy on CT images with an accuracy of 0.88 and an AUC of 0.93 (80).

A meta-analysis examined the SE and SP of DL algorithms and traditional ML approaches for diagnosing COVID-19 from chest X-rays by using studies that reported these measures (81). The inverse variance method and the DerSimonian-Laird estimator were utilized to synthesize the results and evaluate the heterogeneity. The studies exhibited very high heterogeneity (I2 = 98.1% [97.7%; 98.4%], p < 0.0001). The DL algorithms demonstrated significantly higher SE (98.9% [98.7%; 99.1%]) and SP (97.5% [96.9%; 97.9%]) than the traditional ML approaches (p < 0.0001), indicating better diagnostic performance.

The random-effects model indicated that deep learning algorithms had a much higher odds ratio (OR) of 56.2459 [95% CI, 28.3682; 111.5195) and p-value <0.0001 for gynecologic cancer detection than machine learning algorithms, meaning that they were about 56 times more likely to make a correct diagnosis. This is a larger OR than the one found for COVID-19 detection from chest X-rays, which was 9.8 [95% CI, 6.1; 15.7] and p-value <0.0001, meaning that deep learning algorithms were about 10 times more likely to make a correct diagnosis than machine learning algorithms (82). This suggests that deep learning algorithms had a bigger edge over machine learning algorithms for gynecologic cancer detection than for COVID-19 detection.

The diagnostic accuracy of advanced deep learning models for gynecologic cancer was assessed by pooling the sensitivity, specificity, and SROC curve from different studies. The AUC of the SROC curve was 0.81, which indicates a moderate level of accuracy. This is lower than the AUC of 0.86 reported for deep learning models for COVID-19 detection from chest X-rays (83), suggesting that gynecologic cancer diagnosis is more challenging and requires further improvement of the algorithms.

## 5 Conclusion

This review demonstrates that deep learning techniques have been widely utilized in various imaging modalities for detecting, segmenting, and diagnosing gynecologic cancers. Medical image analysis, including lesion segmentation, classification, detection, and quantification, has exhibited tremendous potential with the use of deep learning. The studies reviewed in this paper employed imaging modalities such as cytology, colposcopy, MRI, CT scan, and hysteroscopy. Cytology is utilized for cervical cancer screening and diagnosis by examining cells or tissue fragments under a microscope, while colposcopy inspects the cervix with a magnifying device and is typically done after an abnormal cytology result. MRI and CT scans are non-invasive techniques used to visualize the structure and function of gynecologic organs, while hysteroscopy views the inside of the uterus with a thin camera. These imaging modalities aid in the diagnosis of cervical cancer and other gynecologic cancers, such as endometrial cancer and ovarian cancer. However, they also have drawbacks, including subjectivity, error, low resolution, noise, artifact, and variability. Deep learning can assist cytologists, colposcopists, radiologists, and gynecologists in overcoming these challenges by improving the accuracy, efficiency, objectivity, quality, segmentation, and interpretation of these images.

Normalization, rotation, cropping, and filtering were the most commonly used pre-processing techniques. Max pooling, principal component analysis, and progressive resizing were the most frequently utilized feature extraction techniques. These methods can help achieve higher accuracy, efficiency, and objectivity in diagnosing and prognosing gynecologic cancers using various types of images. However, there is no universal or optimal set of techniques for different imaging modalities, settings, and objectives. As a result, it is critical to choose and customize these methods according to the specific needs and challenges of each application.

The review indicates that neural network architectures such as 3D-UNet, 3D VB-Net, ResNet18, 2D-RefineNet, CE-Net, or fully convolutional neural networks have been frequently utilized for image segmentation and classification, achieving high performance.

## 6 Implication and limitations of the study

Our review demonstrates that CNNs and their variants are effective in detecting gynecologic cancer using medical images, which can aid in diagnosis and treatment. However, our study has some limitations that need to be addressed. First, we did not investigate other types of cancer like breast, lung which are highly prevalent and the review also includes only few imaging modalities so, it lacks the generalizability issue. Since the review focused on comparing the performance of different machine learning and deep learning models, we don’t know the performance of these models as compared with human experts. The summary roc curve of the review indicates that DL algorithms surpass traditional ML approaches in diagnosing gynecologic cancer using medical images. This implies that DL algorithms can improve the early detection and treatment of these diseases, particularly in resource-limited environments where imaging is more feasible than other modalities. However, the diagnostic accuracy of DL algorithms for gynecologic cancer is moderate and requires further improvement. This suggests that there are still challenges and limitations in implementing DL algorithms for this complex and diverse disease, and that additional research is necessary to optimize these algorithms’ performance and generalizability. Therefore, future research should concentrate on developing more robust, dependable, transparent, and ethical deep learning models and techniques for diagnosing gynecologic cancer.

## Author contributions

AT conceived the study, designed the search strategy, performed the literature review, data extraction, quality assessment, and meta-analysis. He also drafted the manuscript and approved the final version. BT conceived the study, designed the search strategy, performed the literature review, data extraction, quality assessment, and meta-analysis. He also revised the manuscript and approved the final version. TA contributed to the data extraction, quality assessment, and meta-analysis. He also revised the manuscript and approved the final version. AA contributed to the data extraction, quality assessment, and meta-analysis. He also revised the manuscript and approved the final version. AM revised the manuscript critically for important intellectual content. He also approved the final version. SAM revised the manuscript critically for important intellectual content. He also approved the final version. All authors contributed to the article and approved the submitted version.

## References

[1] Arnold M Moore SP Hassler S Ellison-Loschmann L Forman D Bray F. The burden of stomach cancer in indigenous populations: a systematic review and global assessment. Gut (2014) 63(1):64–71. doi: 10.1136/gutjnl-2013-305033 24153248

[2] Ferlay J Soerjomataram I Dikshit R Eser S Mathers C Rebelo M. Cancer incidence and mortality worldwide: sources, methods and major patterns in GLOBOCAN 2012. Int J cancer (2015) 136(5):E359–E86. doi: 10.1002/ijc.29210 25220842

[3] Sung H Ferlay J Siegel RL Laversanne M Soerjomataram I Jemal A. Global cancer statistics 2020: GLOBOCAN estimates of incidence and mortality worldwide for 36 cancers in 185 countries. CA: Cancer J Clin (2021) 71(3):209–49. doi: 10.3322/caac.21660 33538338

[4] Chen WQ Li H Sun KX Zheng RS Zhang SW Zeng HM. [Report of cancer incidence and mortality in China 2014]. Zhonghua zhong liu za zhi [Chinese J oncology] (2018) 40(1):5–13. doi: 10.3760/cma.j.issn.0253-3766.2018.01.002 29365411

[5] Guo M Xu J Du J. Trends in cervical cancer mortality in China from 1989 to 2018: an age-period-cohort study and Joinpoint analysis. BMC Public Health (2021) 21(1):1329. doi: 10.1186/s12889-021-11401-8 34229639 PMC8259057

[6] Wardle J Robb K Vernon S Waller J. Screening for prevention and early diagnosis of cancer. Am Psychol (2015) 70(2):119. doi: 10.1037/a0037357 25730719

[7] Haralick RM Shapiro LG. Image segmentation techniques. Comput Vision Graphics Image Processing (1985) 29(1):100–32. doi: 10.1016/S0734-189X(85)90153-7

[8] Atun R Jaffray DA Barton MB Bray F Baumann M Vikram B. Expanding global access to radiotherapy. Lancet Oncol (2015) 16(10):1153–86. doi: 10.1016/S1470-2045(15)00222-3 26419354

[9] Brisson M Kim JJ Canfell K Drolet M Gingras G Burger EA. Impact of HPV vaccination and cervical screening on cervical cancer elimination: a comparative modelling analysis in 78 low-income and lower-middle-income countries. Lancet (2020) 395(10224):575–90. doi: 10.1016/S0140-6736(20)30068-4 PMC7043009 32007141

[10] Sharma N Aggarwal LM. Automated medical image segmentation techniques. J Med Physics (2010) 35(1):3. doi: 10.4103/0971-6203.58777 PMC2825001 20177565

[11] Aerts HJ Velazquez ER Leijenaar RT Parmar C Grossmann P Carvalho S. Decoding tumour phenotype by noninvasive imaging using a quantitative radiomics approach. Nat Commun (2014) 5(1):4006. doi: 10.1038/ncomms5006 24892406 PMC4059926

[12] Vallieres M Kay-Rivest E Perrin LJ Liem X Furstoss C Aerts HJ. Radiomics strategies for risk assessment of tumour failure in head-and-neck cancer. Sci Rep (2017) 7(1):10117. doi: 10.1038/s41598-017-10371-5 28860628 PMC5579274

[13] Lambin P Rios-Velazquez E Leijenaar R Carvalho S Van Stiphout RG Granton P. Radiomics: extracting more information from medical images using advanced feature analysis. Eur J Cancer (2012) 48(4):441–6. doi: 10.1016/j.ejca.2011.11.036 PMC4533986 22257792

[14] Coroller TP Grossmann P Hou Y Velazquez ER Leijenaar RT Hermann G. CT-based radiomic signature predicts distant metastasis in lung adenocarcinoma. Radiother Oncol (2015) 114(3):345–50. doi: 10.1016/j.radonc.2015.02.015 PMC4400248 25746350

[15] Guo W Li H Zhu Y Lan L Yang S Drukker K. Prediction of clinical phenotypes in invasive breast carcinomas from the integration of radiomics and genomics data. J Med Imaging (2015) 2(4):041007–. doi: 10.1117/1.JMI.2.4.041007 PMC4718467 26835491

[16] Wang J Kato F Oyama-Manabe N Li R Cui Y Tha KK. Identifying triple-negative breast cancer using background parenchymal enhancement heterogeneity on dynamic contrast-enhanced MRI: a pilot radiomics study. PloS One (2015) 10(11):e0143308. doi: 10.1371/journal.pone.0143308 26600392 PMC4658011

[17] Zhou K Greenspan H Shen D. Deep learning for medical image analysis. London, UK: Academic Press. (2017).

[18] LeCun Y Bengio Y Hinton G. Deep learning. Nature (2015) 521(7553):436–44. doi: 10.1038/nature14539 26017442

[19] Miotto R Wang F Wang S Jiang X Dudley JT. Deep learning for healthcare: review, opportunities and challenges. Briefings Bioinf (2018) 19(6):1236–46. doi: 10.1093/bib/bbx044 PMC6455466 28481991

[20] Fiste O Liontos M Zagouri F Stamatakos G Dimopoulos MA. Machine learning applications in gynecological cancer: A critical review. Crit Rev Oncol/Hematol (2022) 179:103808. doi: 10.1016/j.critrevonc.2022.103808 36087852

[21] Cozzi L Dinapoli N Fogliata A Hsu W-C Reggiori G Lobefalo F. Radiomics based analysis to predict local control and survival in hepatocellular carcinoma patients treated with volumetric modulated arc therapy. BMC cancer (2017) 17:1–10. doi: 10.1186/s12885-017-3847-7 29207975 PMC5718116

[22] Perrin T Midya A Yamashita R Chakraborty J Saidon T Jarnagin WR. Short-term reproducibility of radiomic features in liver parenchyma and liver Malignancies on contrast-enhanced CT imaging. Abdominal Radiol (2018) 43:3271–8. doi: 10.1007/s00261-018-1600-6 PMC6209534 29730738

[23] Leseur J Roman-Jimenez G Devillers A Ospina-Arango JD Williaume D Castelli J. Pre-and per-treatment 18F-FDG PET/CT parameters to predict recurrence and survival in cervical cancer. Radiother Oncol (2016) 120(3):512–8. doi: 10.1016/j.radonc.2016.08.008 27569847

[24] Reuzé S Orlhac F Chargari C Nioche C Limkin E Riet F. Prediction of cervical cancer recurrence using textural features extracted from 18F-FDG PET images acquired with different scanners. Oncotarget (2017) 8(26):43169. doi: 10.18632/oncotarget.17856 28574816 PMC5522136

[25] Gnep K Fargeas A Gutiérrez-Carvajal RE Commandeur F Mathieu R Ospina JD. Haralick textural features on T2-weighted MRI are associated with biochemical recurrence following radiotherapy for peripheral zone prostate cancer. J Magnetic Resonance Imaging (2017) 45(1):103–17. doi: 10.1002/jmri.25335 27345946

[26] Shiradkar R Ghose S Jambor I Taimen P Ettala O Purysko AS. Radiomic features from pretreatment biparametric MRI predict prostate cancer biochemical recurrence: preliminary findings. J Magnetic Resonance Imaging (2018) 48(6):1626–36. doi: 10.1002/jmri.26178 PMC6222024 29734484

[27] Vallières M Freeman CR Skamene SR El Naqa I. A radiomics model from joint FDG-PET and MRI texture features for the prediction of lung metastases in soft-tissue sarcomas of the extremities. Phys Med Biol (2015) 60(14):5471. doi: 10.1088/0031-9155/60/14/547 26119045

[28] Vallières M Laberge S Diamant A El Naqa I. Enhancement of multimodality texture-based prediction models via optimization of PET and MR image acquisition protocols: a proof of concept. Phys Med Biol (2017) 62(22):8536. doi: 10.1088/1361-6560/aa8a49 28872054

[29] Coroller TP Bi WL Huynh E Abedalthagafi M Aizer AA Greenwald NF. Radiographic prediction of meningioma grade by semantic and radiomic features. PloS One (2017) 12(11):e0187908. doi: 10.1371/journal.pone.0187908 29145421 PMC5690632

[30] Lao J Chen Y Li Z-C Li Q Zhang J Liu J. A deep learning-based radiomics model for prediction of survival in glioblastoma multiforme. Sci Rep (2017) 7(1):10353. doi: 10.1038/s41598-017-10649-8 28871110 PMC5583361

[31] Page MJ McKenzie JE Bossuyt PM Boutron I Hoffmann TC Mulrow CD. The PRISMA 2020 statement: an updated guideline for reporting systematic reviews. Syst Rev (2021) 10(1):89. doi: 10.1186/s13643-021-01626-4 33781348 PMC8008539

[32] Whiting PF Rutjes AW Westwood ME Mallett S Deeks JJ Reitsma JB. QUADAS-2: a revised tool for the quality assessment of diagnostic accuracy studies. Ann Internal Med (2011) 155(8):529–36. doi: 10.7326/0003-4819-155-8-201110180-00009 22007046

[33] Higgins JP Thompson SG Deeks JJ Altman DG. Measuring inconsistency in meta-analyses. BMJ Open (2003) 327:7414: 557–560. doi: 10.1136/bmj.327.7414.557 PMC192859 12958120

[34] DerSimonian RLN. Meta-analysis in clinical trials. Controlled Clin trials (1986) 7(3):177–88. doi: 10.1016/0197-2456(86)90046-2 3802833

[35] Bhatt AR Ganatra A Kotecha KJPCS.. Cervical cancer detection in pap smears whole slide images using convnet with transfer learning and progressive resizing. (2021) 7:. 10.7717/peerj-cs.348 PMC7959623 33816998

[36] Chandran V Sumithra M Karthick A George T Deivakani M Elakkiya B. Diagnosis of cervical cancer based on ensemble deep learning network using colposcopy images. (2021) 2021:. 10.1155/2021/5584004 PMC8112909 33997017

[37] Cheng S Liu S Yu J Rao G Xiao Y Han W. Robust whole slide image analysis for cervical cancer screening using deep learning. (2021) 12(1):5639. 10.1038/s41467-021-25296-x PMC8463673 34561435

[38] Chen X Wang Y Shen M Yang B Zhou Q Yi Y. Deep learning for the determination of myometrial invasion depth and automatic lesion identification in endometrial cancer MR imaging: a preliminary study in a single institution. (2020) 30:4985–94. 10.1007/s00330-020-06870-1 32337640

[39] Cho B-J Choi YJ Lee M-J Kim JH Son G-H Park S-H. Classification of cervical neoplasms on colposcopic photography using deep learning. (2020) 10(1):13652. 10.1038/s41598-020-70490-4 PMC7423899 32788635

[40] Dai M Liu Y Hu Y Li G Zhang J Xiao Z. Combining multiparametric MRI features-based transfer learning and clinical parameters: application of machine learning for the differentiation of uterine sarcomas from atypical leiomyomas. (2022) 32(11):7988–97. 10.1007/s00330-022-08783-7 35583712

[41] AbuKhalil T Alqaralleh BA Al-Omari AHJCMC.. Optimal deep learning based inception model for cervical cancer diagnosis. (2022) 72:57–71.

[42] Alquran H Mustafa WA Qasmieh IA Yacob YM Alsalatie M Al-Issa Y. Cervical cancer classification using combined machine learning and deep learning approach. Computers Materials Continua. (2022) 72(3):5117–34.

[43] Best MG Wesseling P Wurdinger T. Tumor-educated platelets as a noninvasive biomarker source for cancer detection and progression monitoring. (2018) 78(13):3407–12. 10.1158/0008-5472.CAN-18-0887 29921699

[44] Cheng WF Chen CA Wang S Liu Z Rong Y Zhou B. Deep learning provides a new computed tomography-based prognostic biomarker for recurrence prediction in high-grade serous ovarian cancer. Cancers (Basel). (2019) 132:171–7. 10.1016/j.radonc.2018.10.019 30392780

[45] Habtemariam LW Zewde ET Simegn GLJMDE, Research. Cervix type and cervical cancer classification system using deep learning techniques. (2022), 163–76. 10.2147/MDER.S366303 PMC9208738 35734419

[46] Kudva V Prasad K Guruvare S. Hybrid transfer learning for classification of uterine cervix images for cervical cancer screening. (2020) 33:619–31. 10.1007/s10278-019-00269-1 PMC7256135 31848896

[47] Ding Y Chen Z Wang Z Wang X Hu D Ma P. Three-dimensional deep neural network for automatic delineation of cervical cancer in planning computed tomography images. (2022) 23(4):. 10.1002/acm2.13566 PMC8992957 35192243

[48] Ma CY Zhou JY Xu XT Guo J Han MF Gao YZ. Deep learning-based auto-segmentation of clinical target volumes for radiotherapy treatment of cervical cancer. (2022) 23(2):. 10.1002/acm2.13470 PMC8833283 34807501

[49] Lin Y Wang Y Li X Zhang Y Wang Y Zhang J. Prostate segmentation in MRI using ResNet18 with multi-scale inputs and outputs. In: 2020 IEEE International Conference on Image Processing (ICIP); October 25 to 28, 2020; Abu Dhabi, United Arab Emirates. Abu Dhabi, UAE: IEEE (2020). pp. 3369–73. doi: 10.1109/ICIP40778.2020.9191238

[50] Williams MS Kenu E Dzubey I Dennis-Antwi JA Fontaine K. A qualitative study of cervical cancer and cervical cancer screening awareness among nurses in ghana. Eur Radiology. (2018) 39(5):584–94. 10.1080/07399332.2018.1424169 29334011

[51] Xiao C Jin J Yi J Han C Zhou Y Ai Y. RefineNet-based 2D and 3D automatic segmentations for clinical target volume and organs at risks for patients with cervical cancer in postoperative radiotherapy. (2022) 23(7):. 10.1002/acm2.13631 PMC9278674 35533205

[52] Zaffino P Pernelle G Mastmeyer A Spadea MF Sharp GC Knopf AC. 3D VB-Net: Volumetric boundary-aware network for ovarian cancer segmentation in MRI. Med Phys (2020) 47(8):3749–61. doi: 10.1002/mp.14237

[53] Cho B-J Kim J-W Park J Kwon G-Y Hong M Jang S-H. Automated diagnosis of cervical intraepithelial neoplasia in histology images via deep learning. Sci. Rep.(2022) 12(2):548. 35204638 10.3390/diagnostics12020548 PMC8871214

[54] Hou X Wang Y Li X Zhang J Wang Y Li Y. Ovarian cancer detection in MRI using EfficientNetB0 with multi-scale inputs and outputs. Biomed Signal Process Control (2022) 70:103251. doi: 10.1016/jbspc2021103251

[55] Karasu Benyes Y Welch EC Singhal A Ou J Tripathi AJD. A comparative analysis of deep learning models for automated cross-preparation diagnosis of multi-cell liquid pap smear images. Diagnostics (2022) 12(8):1838. 36010189 10.3390/diagnostics12081838 PMC9406372

[56] Saini SK Bansal V Kaur R Juneja MJMV, Applications. ColpoNet for automated cervical cancer screening using colposcopy images. Mach Vision App (2020) 31:1–15.

[57] Nambu Y Mariya T Shinkai S Umemoto M Asanuma H Sato I. A screening assistance system for cervical cytology of squamous cell atypia based on a two-step combined CNN algorithm with label smoothing. Cancer Med (2022) 11(2):520–9. 10.1002/cam4.4460 PMC8729059 34841722

[58] Park YR Kim YJ Ju W Nam K Kim S Kim KGJSR.. Comparison of machine and deep learning for the classification of cervical cancer based on cervicography images. Sci Rep (2021) 11(1):16143. 34373589 10.1038/s41598-021-95748-3 PMC8352876

[59] Sheikhzadeh F Ward RK van Niekerk D Guillaud M. Automatic labeling of molecular biomarkers of immunohistochemistry images using fully convolutional networks. PloS one (2018) 13(1):. 10.1371/journal.pone.0190783 PMC5774709 29351281

[60] Wang H Jiang C Bao K Xu C. Recognition and clinical diagnosis of cervical cancer cells based on our improved lightweight deep network for pathological image. J Cell Mol Med (2019) 43:1–9. 10.1007/s10916-019-1426-y 31372766

[61] Kudva V Prasad K Guruvare S eds. Transfer learning for classification of uterine cervix images for cervical cancer screening. In: Advances in communication, signal processing, VLSI, and embedded systems: Select proceedings of VSPICE 2019. Springer.

[62] Sun H Zeng X Xu T Peng G Ma Y. Computer-aided diagnosis in histopathological images of the endometrium using a convolutional neural network and attention mechanisms. J Healthcare Engineering. (2020) 24(6):1664–76. 10.1109/JBHI.2019.2944977 31581102

[63] Urushibara A Saida T Mori K Ishiguro T Inoue K Masumoto T. The efficacy of deep learning models in the diagnosis of endometrial cancer using MRI: a comparison with radiologists. BMC Med Imaging (2022) 22(1):1–14. 35501705 10.1186/s12880-022-00808-3 PMC9063362

[64] Zhang T Zhang X Ke X Liu C Xu X Zhan X. HOG-ShipCLSNet: A novel deep learning network with hog feature fusion for SAR ship classification. IEEE Trans Geosci Remote Sensing (2021) 60:1–22. doi: 10.1109/TGRS.2021.3061417

[65] Takahashi Y Sone K Noda K Yoshida K Toyohara Y Kato K. Automated system for diagnosing endometrial cancer by adopting deep-learning technology in hysteroscopy. PLoS One (2021) 16(3):. 10.1371/journal.pone.0248526 PMC8011803 33788887

[66] Tong Y Lu W Deng Q-Q Chen C Shen Y. Automated identification of retinopathy of prematurity by image-based deep learning. Eye Vision (2020) 7:40. doi: 10.1186/s40662-020-00206-2 32766357 PMC7395360

[67] Daoud T Sardana S Stanietzky N Klekers AR Bhosale P Morani AC. Recent imaging updates and advances in gynecologic Malignancies. Cancers (2022) 14(22):5528. doi: 10.3390/cancers14225528 36428624 PMC9688526

[68] Khan SR Arshad M Wallitt K Stewart V Bharwani N Barwick TD. What’s new in imaging for gynecologic cancer? Curr Oncol Rep (2017) 19(12):85. doi: 10.1007/s11912-017-0640-3 29105030

[69] Bragg DG Hricak H. Imaging in gynecologic Malignancies. Cancer (1993) 71(S4):1648–51. doi: 10.1002/cncr.2820710431 8431900

[70] Yadav BK Panse M. Different image pre-processing and feature extraction techniques for breast cancer detection in labview. Int J Comput Appl (2016) 147(10):1–6. doi: 10.5120/ijca2016911752

[71] Torres-García AA Mendoza-Montoya O Molinas M Antelis JM Moctezuma LA Hernández-Del-Toro T. Chapter 4 - Pre-processing and feature extraction. In: Torres-García AA Reyes-García CA Villaseñor-Pineda L Mendoza-Montoya O, editors. Biosignal processing and classification using computational learning and intelligence. Cambridge, MA, USA: Academic Press. (2022). p. 59–91.

[72] Kalbhor M Shinde S Popescu DE Hemanth DJ. Hybridization of deep learning pre-trained models with machine learning classifiers and fuzzy min&ndash;Max neural network for cervical cancer diagnosis. Diagnostics (2023) 13(7):1363. doi: 10.3390/diagnostics13071363 37046581 PMC10093705

[73] Woo J Xing F Prince JL Stone M Gomez AD Reese TG. A deep joint sparse non-negative matrix factorization framework for identifying the common and subject-specific functional units of tongue motion during speech. Med image Anal (2021) 72:102131. doi: 10.1016/j.media.2021.102131 34174748 PMC8316408

[74] Pastor-Serrano O Lathouwers D Perkó Z. A semi-supervised autoencoder framework for joint generation and classification of breathing. Comput Methods Programs Biomed (2021) 209:106312. doi: 10.1016/j.cmpb.2021.106312 34392000

[75] Guruvare S Bhatt A Chaudhary A Kothari A Rangarajan A Kothari R. Cervical cancer detection using convolutional neural networks with transfer learning and progressive resizing. J Med Imaging Health Inf (2021) 11(4):1129–36. doi: 10.1166/jmihi.2021.3459

[76] Cao X Chen H Li Y Peng Y Wang S Cheng L. Dilated densely connected U-Net with uncertainty focus loss for 3D ABUS mass segmentation. Comput Methods Programs Biomed (2021) 209:106313. doi: 10.1016/j.cmpb.2021.106313 34364182

[77] Tufail AB Ma YK. Deep learning in cancer diagnosis and prognosis prediction: A minireview on challenges. Recent Trends Future Dir (2021) 2021:9025470. doi: 10.1155/2021/9025470 PMC8572604 34754327

[78] Gao Y Zeng S Xu X Li H Yao S Song K. Deep learning-enabled pelvic ultrasound images for accurate diagnosis of ovarian cancer in China: a retrospective, multicentre, diagnostic study. Lancet Digital Health (2022) 4(3):e179–e87. doi: 10.1016/S2589-7500(21)00278-8 35216752

[79] Li Z Zhu Q Zhang L Yang X Li Z Fu J. A deep learning-based self-adapting ensemble method for segmentation in gynecological brachytherapy. Radiat Oncol (2022) 17(1):152. 36064571 10.1186/s13014-022-02121-3 PMC9446699

[80] Akazawa M Hashimoto K. Artificial intelligence in gynecologic cancers: Current status and future challenges - A systematic review. Artif Intell Med (2021) 120:102164. doi: 10.1016/j.artmed.2021.102164 34629152

[81] Wang S Zha Y Li W Wu Q Li X Niu M. A fully automatic deep learning system for COVID-19 diagnostic and prognostic analysis. Eur Respir J (2020) 56(2):2000775. doi: 10.1183/13993003.00775-2020 32444412 PMC7243395

[82] Passalis N Tefas A Kanniainen J Gabbouj M Iosifidis A. Temporal logistic neural bag-of-features for financial time series forecasting leveraging limit order book data. Pattern Recognit Lett (2020) 136:183–9. doi: 10.1016/j.patrec.2020.06.006

[83] Bae S An C Ahn SS Kim H Han K Kim SW. Robust performance of deep learning for distinguishing glioblastoma from single brain metastasis using radiomic features: model development and validation. Sci Rep (2020) 10(1):1–10. doi: 10.1038/s41598-020-68980-6 32694637 PMC7374174

