# Supplementary material for: Deep-learning models for image-based gynecological cancer diagnosis: a systematic review and meta- analysis
Source: Front Oncol. 2024 Jan 11;13:1216326. doi: 10.3389/fonc.2023.1216326 (PMC10809847; doi:10.3389/fonc.2023.1216326)
Supplement: Supplementary file 1 [file DataSheet_1.zip › Supplementary file 1.docx]

**Supplementary file 1: Deep learning models used by extracted articles**

| Authors | Year of publication | Number of images | Deep learning models |
| --- | --- | --- | --- |
| AbuKhalil, T., et al.([35](#_ENREF_35)) | 2022 | 918 | Inception ResNet- v2 |
|  |  |  | Ensemble Learning |
|  |  |  | Recurrent Neural Network (RNN) |
| Alquran, H., et al.([36](#_ENREF_36)) | 2022 | 917 | Ensemble Support Vector Machine (SVM) |
| Best, M. G., et al.([37](#_ENREF_37)) | 2021 | 7530 | ResNet-50 |
| Bhatt AR.et al  ([38](#_ENREF_38)) | 2021 | 917 | K-Convolutional Nearest Neighbors(K-CNN) |
|  |  |  | Ensemble Support Vector Machine (SVM) |
|  |  |  | Convolution Random Forest |
|  | 2021 | 917 | VGG19 |
|  |  |  | ResNet-34 |
|  |  |  | ResNet-101 |
|  |  |  | EfficientNetB3 |
|  |  |  | EfficientNetB4 |
|  |  |  | EfficientNetB5 |
|  | 2021 | 966 | VGG19 |
|  |  |  | ResNet-34 |
|  |  |  | ResNet-101 |
|  |  |  | EfficientNetB3 |
| Chandran, V., et al.([39](#_ENREF_39)) | 2021 | 5679 | DenseNet-121 |
|  |  |  | DenseNet-169 |
|  |  |  | ColpNet |
|  |  |  | Ensemble Support Vector Machine (SVM) |
|  |  |  | Inception-Resnet-v2 |
|  |  |  | CYENET |
|  |  |  | VGG19 |
| Chang, Y., et al.([40](#_ENREF_40)) | 2021 | 400 | 3D-UNet |
| Chen, X., et al. ([41](#_ENREF_41)) | 2020 | 530 | You Only Look Once v3 (YOLOWv3) |
| Cheng, S., et al.([42](#_ENREF_42)) | 2021 | 3545 | Recurrent Neural Network (RNN) |
| Cheng, W. F., et al.([43](#_ENREF_43)) | 2019 | 84 | ensemble Support Vector Machine (SVM) |
| Cho, B. J., et al. ([44](#_ENREF_44)) | 2020 | 791 | Inception-Resnet-v2 |
|  |  |  | Resnet-152 |
| Cho, B. J., et al.([45](#_ENREF_45)) | 2020 | 588 | Inception-Resnet-v2 |
|  |  |  | Resnet-152 |
|  |  |  | DenseNet-161 |
|  |  |  | EfficienetNetB0 |
| Dai, M., et al.([46](#_ENREF_46)) | 2022 | 86 | 3D-UNet |
|  |  |  | InceptionV3 |
|  |  |  | ResNet-50 |
|  |  |  | Inception-Resnet-v2 |
|  |  |  | Xception |
| Ding, Y., et al.([47](#_ENREF_47)) | 2022 | 130 | 3D-UNet |
|  |  |  | 3D VB-Net |
| Guruvare, S., et al.([48](#_ENREF_48)) | 2021 | 2198 | Alex Net |
|  |  |  | VGG-16 |
|  |  |  | Fully Convolutional Neural Network (FCNN) |
| Habtemariam, L. W., et al.([49](#_ENREF_49)) | 2022 | 4005 | ResNet-50 |
|  |  |  | VGG-16 |
|  |  |  | Hybrid ResNet50 and MobilenetV2 |
|  |  |  | EfficienetNetB0 |
| Hou, X., et al.([50](#_ENREF_50)) | 2022 | 34 | VGG-16 |
|  |  |  | Hybrid ResNet50 and MobilenetV2 |
|  |  |  | EfficienetNetB0 |
| Song et al. ([51](#_ENREF_51)) | 2019 | 22 | Fully Convolutional Neural Network (FCNN) |
| Huttunen et al.([52](#_ENREF_52)) | 2018 | 200 | Alex Net |
|  |  |  | VGG-16 |
|  |  |  | VGG19 |
|  |  |  | Google Net |
| Hu, L., et al.([53](#_ENREF_53)) | 2019 | 9406 | Fully Convolutional Neural Network (FCNN) |
| Xiangyu M. et al([54](#_ENREF_54)) | 2020 | 400 | UNet2 |
|  |  |  | UNet4 |
|  |  |  | UNet6 |
| Kanavati, F., et al.([55](#_ENREF_55)) | 2022 | 3121 | Hybrid Convolutional Neural Network (CNN) and Recurrent Neural Network (RNN) |
| Karasu Benyes, Y., et al.([56](#_ENREF_56)) | 2022 | 2452 | Fully Convolutional Neural Network (FCNN) |
|  |  |  | Auto Encoder (AE) |
|  |  |  | ResNet-50 |
|  |  |  | DenseNet-121 |
|  |  |  | ResNet-101 |
|  |  |  | ResNet152 |
|  |  |  | EfficienetNetB0 |
| Ke, J. and Y. Shen([57](#_ENREF_57)) | 2021 | 130 | U-Net |
|  |  |  | Recurrent Neural Network (RNN) |
|  |  |  | 3D-UNet |
|  |  |  | Alex Net |
|  |  |  | VGG-16 |
|  |  |  | ResNet-50 |
|  |  |  | Inception-Resnet-v2 |
| Kim, Y. J., et al.([58](#_ENREF_58)) | 2022 | 670 | ResNet-50 |
|  |  | 438 | ResNet-50 |
|  |  | 477 | ResNet-50 |
| Kudva, R., et al.([59](#_ENREF_59)) | 2020 | 749 | Fully Convolutional Neural Network (FCNN) |
| Kudva, V., et al([60](#_ENREF_60)) | 2020 | 2198 | Alex Net |
|  |  |  | VGG-16 |
|  |  |  | Fully Convolutional Neural Network (FCNN) |
|  |  |  | Ensemble learning |
| Lin, Y.C., et al.([61](#_ENREF_61)) | 2020 | 169 | ResNet18 |
| Ma, C. Y., et al.([62](#_ENREF_62)) | 2022 | 200 | 3D VB-Net |
|  |  | 335 | 3D VB-Net |
|  |  | 302 | 3D VB-Net |
|  |  | 107 | 3D VB-Net |
| Ma, J., et al. ([63](#_ENREF_63)) | 2021 | 156 | Convolutional Logistic Regression |
|  |  |  | Ensemble Gradient Boosting |
|  |  |  | Conditional Random Forest (CRF) |
|  |  |  | Ensemble Support Vector Machine (SVM) |
| Maruyama, M., et al. ([64](#_ENREF_64)) | 2021 | 67811 | X-caption |
|  |  | 177 | Mobile Net v2 |
|  |  |  | EfficienetNetB0 |
| Mix, J. M., et al.([65](#_ENREF_65)) | 2022 | 5679 | CYENET |
|  |  |  | VGG19 |
| Saini, Bansal et al.([66](#_ENREF_66)) | 2020 | 400 | ColpNet |
| Xu, Zhang et al.([67](#_ENREF_67)) | 2017 |  | Ensemble Support Vector Machine (SVM) |
| Nambu, Y. et al.([68](#_ENREF_68)) | 2022 | 919 | You Only Look Once v4 (YOLOv4) |
|  |  |  | ResNeSt |
| Park, Y. R., et al.([69](#_ENREF_69)) | 2021 | 4119 | Ensemble Extreme gradient boosting (XGBoost) |
|  |  |  | Ensemble Support Vector Machine (SVM) |
|  |  |  | Convolutional Random Forest |
|  |  |  | ResNet-50 |
| Reilly, G. et,al([70](#_ENREF_70)) | 2022 | 1067 | MIA3G |
| Sengupta, Ali et al. ([71](#_ENREF_71)) | 2022 | 485 | Ensemble learning |
| Sheikhzadeh, F., et al.([72](#_ENREF_72)) | 2018 | 749 | Fully convolutional networks (FCNs) |
| Sun, H., et al.([73](#_ENREF_73)) | 2020 | 500 | HIENet |
| Urushibara, A., et al.([74](#_ENREF_74)) | 2022 | 485 | Fully convolutional networks (FCNs) |
| Wang, H., et al.([75](#_ENREF_75)) | 2022 | 917 | ResNet-50 |
|  |  |  | DResNet |
|  |  |  | ResNet18 |
|  |  |  | ShuffleNet |
|  |  |  | MobileNet |
|  |  |  | MobileNet v2 |
|  |  |  | DResNet |
| Williams, M., et al. ([76](#_ENREF_76)) | 2018 | 169 | 3D-UNet |
| Xiao, C., et al.([77](#_ENREF_77)) | 2022 | 313 | 2D-RefineNet |
|  |  |  | Fully convolutional networks (FCNs) |
|  |  |  | 2D-UNet |
|  |  |  | Context Encoder Network (CE-Net) |
|  |  |  | 3D-UNet |
|  |  |  | 3D- ResUNet |
|  |  |  | 3D-RefineNet |
| Zaffino, P., et al.([78](#_ENREF_78)) | 2019 | 826 | 3D-UNet |
| Zhang, L., et al.([79](#_ENREF_79)) | 2019 | 428 | VGGNet |
|  |  |  | Alex Net |
|  |  |  | Fully convolutional networks (FCNs) |
|  |  |  | Inception V1/ Google Net |
|  |  |  | Cost-sensitive random forest |
| Zhang, Y. Z., et al.([80](#_ENREF_80)) | 2021 | 1851 | VGG-16 |
