# Supplementary material for: Deep-learning models for image-based gynecological cancer diagnosis: a systematic review and meta- analysis
Source: Front Oncol. 2024 Jan 11;13:1216326. doi: 10.3389/fonc.2023.1216326 (PMC10809847; doi:10.3389/fonc.2023.1216326)
Supplement: Supplementary file 1 [file DataSheet_1.zip › Supplementary file 2.PDF]

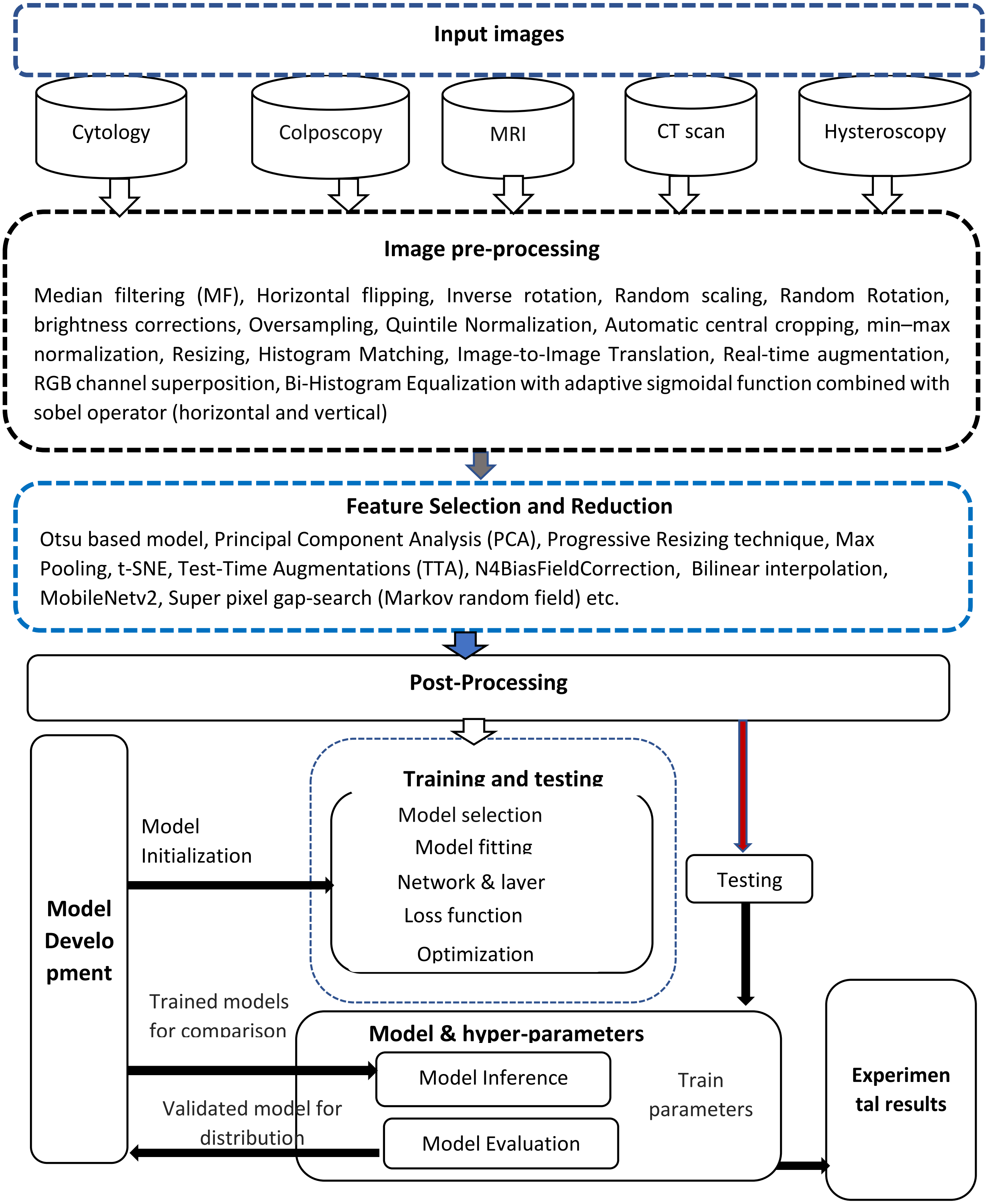

Supplementary file 2: *Shows the schematic flow of deep learning models for image based gynecologic cancer based on different articles in this review*
