# Supplementary material for: Deep-learning models for image-based gynecological cancer diagnosis: a systematic review and meta- analysis
Source: Front Oncol. 2024 Jan 11;13:1216326. doi: 10.3389/fonc.2023.1216326 (PMC10809847; doi:10.3389/fonc.2023.1216326)
Supplement: Supplementary file 1 [file DataSheet_1.zip › Supplementary file 3.PDF]

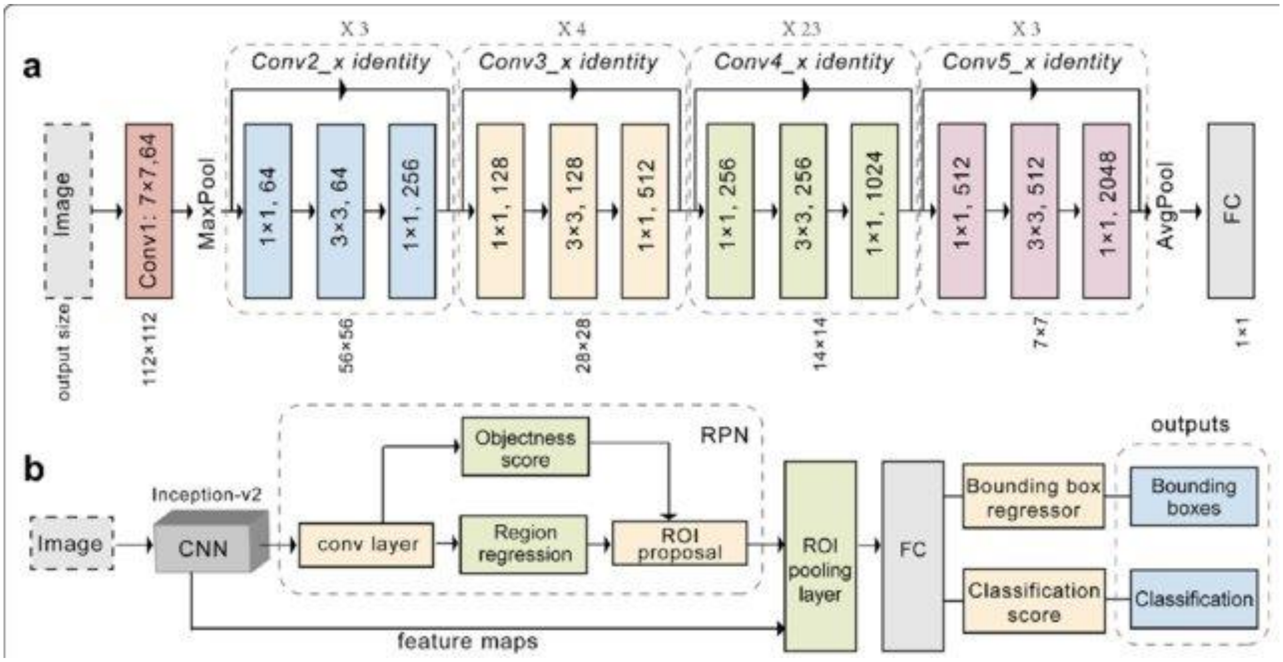

Supplementary file 3: presents a workflow diagram with two key components: a. It illustrates the typical architecture of a 101-layer ResNet, a deep neural network widely used for image classification. b. The flowchart depicts Faster-RCNN, including various components such as Conv (convolutional layer), RPN (region proposal network), ROI (region of interest), AvgPool (average pool), FC (fully connected layer), and CN (35).
